# LincRNA‐EPS impairs host antiviral immunity by antagonizing viral RNA–PKR interaction

**DOI:** 10.15252/embr.202153937

**Published:** 2022-03-21

**Authors:** Jingfei Zhu, Shengchuan Chen, Li‐Qiong Sun, Siying Liu, Xue Bai, Dapei Li, Fan Zhang, Zigang Qiao, Liang Li, Haiping Yao, Yu Xia, Ping Xu, Xiaohui Jiang, Zhengrong Chen, Yongdong Yan, Feng Ma

**Affiliations:** ^1^ CAMS Key Laboratory of Synthetic Biology Regulatory Elements Institute of Systems Medicine Chinese Academy of Medical Sciences & Peking Union Medical College Beijing China; ^2^ Suzhou Institute of Systems Medicine Suzhou China; ^3^ Department of Hepatopancreatobiliary Surgery The First Affiliated Hospital of Wenzhou Medical University Wenzhou China; ^4^ Institute of Chinese Medicinal Materials Nanjing Agricultural University Nanjing China; ^5^ Suzhou Center for Disease Control and Prevention Suzhou China; ^6^ Department of Laboratory Medicine The Fifth People’s Hospital of Suzhou Suzhou China; ^7^ Department of Pulmonary Medicine Children’s Hospital of Soochow University Suzhou China

**Keywords:** lncRNA, lincRNA‐EPS, type I interferon, antiviral immunity, PKR, Immunology, Microbiology, Virology & Host Pathogen Interaction, RNA Biology

## Abstract

LincRNA‐EPS is an important regulator in inflammation. However, the role of lincRNA‐EPS in the host response against viral infection is unexplored. Here, we show that lincRNA‐EPS is downregulated in macrophages infected with different viruses including VSV, SeV, and HSV‐1. Overexpression of lincRNA‐EPS facilitates viral infection, while deficiency of lincRNA‐EPS protects the host against viral infection *in vitro* and *in vivo*. *LincRNA‐EPS* ^−/−^ macrophages show elevated expression of antiviral interferon‐stimulated genes (ISGs) such as *Mx1, Oas2,* and *Ifit2* at both basal and inducible levels. However, IFN‐β, the key upstream inducer of these ISGs, is downregulated in *lincRNA‐EPS* ^−/−^ macrophages compared with control cells. RNA pulldown and mass spectrometry results indicate that lincRNA‐EPS binds to PKR and antagonizes the viral RNA–PKR interaction. PKR activates STAT1 and induces antiviral ISGs independent of IFN‐I induction. LincRNA‐EPS inhibits PKR‐STAT1‐ISGs signaling and thus facilitates viral infection. Our study outlines an alternative antiviral pathway, with downregulation of lincRNA‐EPS promoting the induction of PKR‐STAT1‐dependent ISGs, and reveals a potential therapeutic target for viral infectious diseases.

## Graphical abstract

The lncRNA lincRNA‐EPS binds to PKR in the cytoplasm of macrophages, antagonizes viral RNA‐PKR interaction, and thus impairs PKR‐STAT1‐dependent host antiviral immunity.

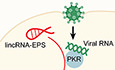

## Introduction

The innate immune system is the first line of defense against pathogenic microbes including numerous life‐threatening viruses. During viral infection, viral RNA or DNA are recognized by pattern recognition receptors (PRRs) to initiate complex signal transduction pathways, which ultimately leads to the induction of type I interferon (IFN‐I) and proinflammatory cytokines (Akira *et al*, 2006; Goubau *et al*, 2013). Retinoic acid‐inducible gene I (RIG‐I) is one of the key cytosolic RNA sensors that recognize viral RNA from invaded viruses (Yoneyama *et al*, 2004). Viral RNA‐triggered activation of RIG‐I results in the phosphorylation of TBK1 and IRF3, which activates transcription factors including NF‐κB, AP‐1, and IRF3/7 to induce proinflammatory cytokines and IFN‐I (Honda *et al*, 2006). IFN‐I including IFN‐α and IFN‐β further trigger the phosphorylation of transcription factors STAT1 and STAT2 via the JAK‐STAT pathway to induce multiple IFN‐stimulated genes (ISGs) such as *MX1*, *OAS2*, *ISG15,* and *IFIT2*, which synergistically inhibit viral infection by targeting almost all the steps of viral life cycles (Sadler & Williams, 2008; Schneider *et al*, 2014). However, overproduction of IFN‐I and hyperactivation of IFN‐α/β receptor (IFNAR) downstream signaling lead to autoimmune diseases including systemic lupus erythematosus (SLE) and Aicardi‐Goutières syndrome (AGS) (Chaussabel *et al*, 2008; Crow & Manel, 2015). The innate immune signaling cascades during viral infection are precisely controlled by various negative feedback pathways, which protect the host by efficiently clearing invaded pathogens but avoiding autoimmunity (Wang *et al*, 2017b; Vierbuchen & Fitzgerald, 2021).

In addition to RIG‐I, PKR is well‐known as a nucleic acids receptor of viral dsRNA produced from replication or transcription intermediates of a wide range of virus families such as negative‐strand RNA viruses VSV and Sendai virus (SeV) (Stojdl *et al*, 2000; Dauber & Wolff, 2009), positive‐strand RNA virus Hepatitis C virus (Targett‐Adams *et al*, 2008), and DNA virus Herpes simplex virus type 1 (HSV‐1) (Jacquemont & Roizman, 1975). PKR is autophosphorylated and activated following sensing viral dsRNA and then phosphorylates eukaryotic translation initiation factor 2 on its α subunit (eIF2α) to inhibit translation initiation of viral proteins (Dalet *et al*, 2015). Cellular non‐coding RNAs, such as the inverted Alu repeats (IRAlus) elements located in the 3′‐untranslated regions (3′‐UTR) and mitochondrial RNAs (mtRNAs) formed intermolecular dsRNA, also activate PKR through direct interaction to regulate cellular proliferation or metabolism (Kim *et al*, 2014, 2018). In addition to be activated by the cellular RNA, PKR is suppressed during binding with the cytoplasmic circular RNAs (circRNAs) that tend to form 16–26 bp imperfect RNA duplexes, and viral infection relieves this inhibition following circRNAs degradation by RNase L to activate PKR activity (Liu *et al*, 2019). However, it is unclear whether any linear long non‐coding RNAs (lncRNAs) regulate PKR‐dependent antiviral immunity.

PKR is also required for the activation of MAPK and IKK complex, as well as the transcriptional activities of IRF1 and STAT1 (Wong *et al*, 1997; Garcia *et al*, 2006; Gal‐Ben‐Ari *et al*, 2018). As an IFN‐I inducible gene, PKR directly associates with STAT1 via the PKR dsRNA‐binding domain (Tanaka & Samuel, 1994). PKR is essential for the phosphorylation of STAT1 on Ser727 and Tyr701 under the response to IFN‐γ and LPS (Ramana *et al*, 2000; Lee *et al*, 2005; Karehed *et al*, 2007). LncRNA *GRASLND* acts to inhibit IFN‐γ signaling by binding PKR and in turn inhibiting STAT1 activity during chondrogenesis (Huynh *et al*, 2020). However, it is unexplored whether any PKR‐interacted lncRNAs contribute to the regulation of the IFN‐I‐JAK‐STAT pathway during host innate immunity against viral infection.

The long intergenic noncoding RNA lincRNA‐EPS was initially reported to inhibit apoptosis during erythroid cell differentiation in part through repressing the expression of the proapoptotic gene Pycard (Hu *et al*, 2011). During inflammatory responses, lincRNA‐EPS is tightly regulated in macrophages to control the expression of immune response genes (IRGs) at the transcription level by interacting with hnRNPL (Atianand *et al*, 2016). The deficiency of lincRNA‐EPS enhances inflammatory response and leads to death in the endotoxin‐shock mouse model while protecting the host from *Listeria monocytogenes* infection (Atianand *et al*, 2016; Agliano *et al*, 2019). Furthermore, knockdown of lincRNA‐EPS promoted autophagy in Bacillus Calmette‐Guérin (BCG)‐infected RAW264.7 macrophages by activating the JNK/MAPK pathway, and the downregulation of lincRNA‐EPS was shown in active pulmonary tuberculosis (PTB) patients (Ke *et al*, 2020). Our previous study has described that lincRNA‐EPS alleviates severe acute pancreatitis by suppressing HMGB1‐triggered inflammation in pancreatic macrophages (Chen *et al*, 2021). Although lincRNA‐EPS has been well identified as a key immunoregulatory lncRNA that restrains inflammatory responses, the role of lincRNA‐EPS in antiviral immunity was not yet studied.

In this study, we have found that the expression of lincRNA‐EPS is also precisely controlled during host antiviral immunity in IFN‐I‐ and NF‐κB‐dependent manners. Downregulation of lincRNA‐EPS protects the host against viral infection *in vitro* and *in vivo*. LincRNA‐EPS binds to PKR and thus negatively regulates PKR‐STAT1‐dependent host antiviral immunity by antagonizing the interaction between viral RNA and PKR. Our study has indicated that lincRNA‐EPS plays an important role in modulating host innate antiviral immunity.

## Results

### Downregulation of lincRNA‐EPS by host antiviral immunity

**Figure 1:**
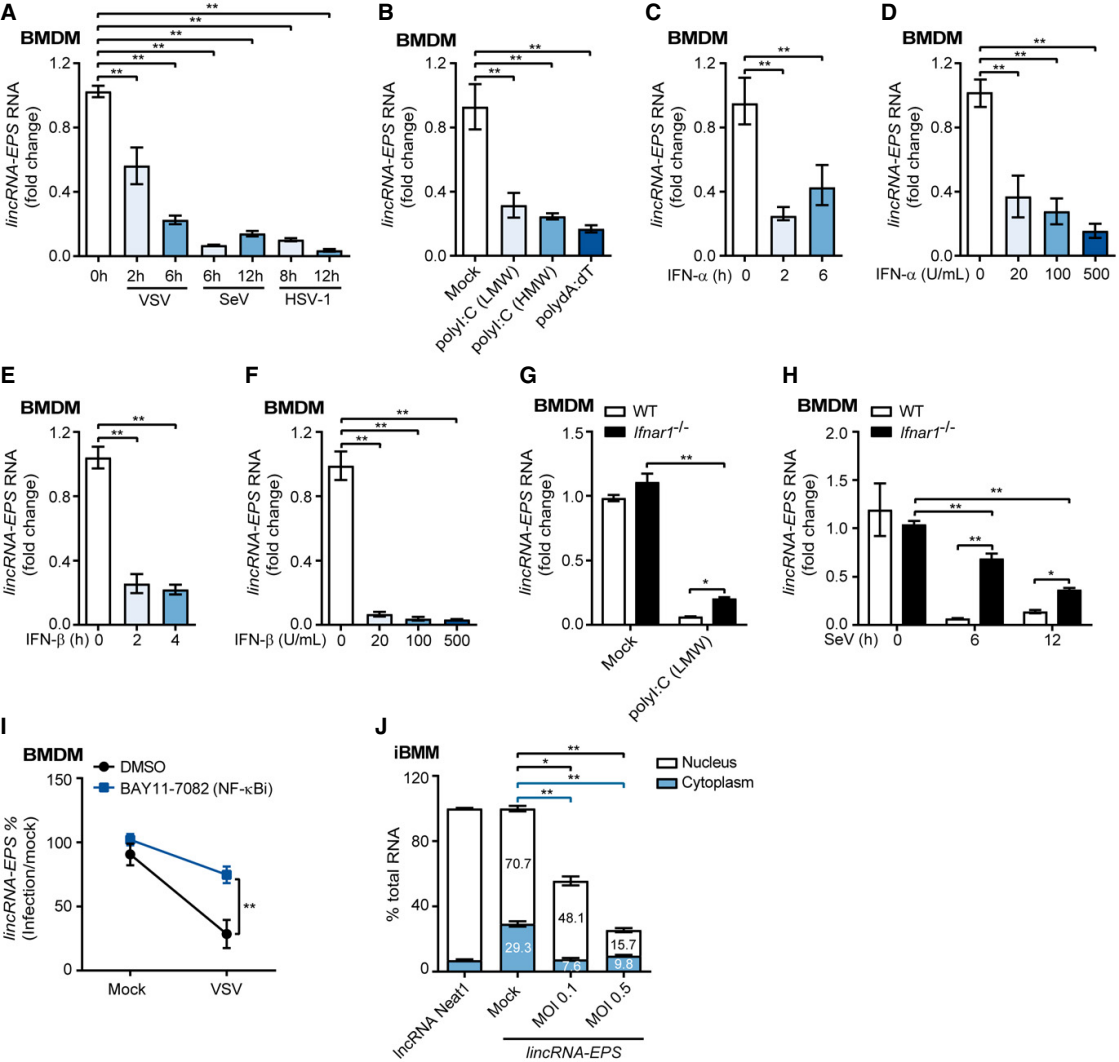
Downregulation of lincRNA‐EPS by host antiviral immunity ART–qPCR analysis of lincRNA‐EPS transcripts in the BMDMs infected with VSV (MOI 1), SeV (MOI 1), or HSV‐1 (MOI 5) for indicated time points.BRT–qPCR analysis of lincRNA‐EPS transcripts in the BMDMs transfected with 1 μg/ml low molecular weight (LMW), high molecular weight (HMW) polyI:C, and polydA:dT for 6 h.C, DRT–qPCR analysis of lincRNA‐EPS transcripts in the BMDMs stimulated with 500 U/ml IFN‐α for different time points (C) or different concentrations for 2 h (D).E, FRT–qPCR analysis of lincRNA‐EPS transcripts in the BMDMs stimulated with 500 U/ml IFN‐β for different time points (E) or different concentrations for 2 h (F).G, HRT–qPCR analysis of lincRNA‐EPS transcripts in the WT and *Ifnar1* ^−/−^ BMDMs transfected with 1 μg/ml polyI:C (LMW) for 6 h (G) and infected with SeV (MOI 1) for indicated time points (H).IBMDMs were pretreated with NF‐κB inhibitor BAY11‐7082 (1 μM) for 1 h and infected with VSV (MOI 1) for 6 h. The percentage of lincRNA‐EPS transcripts downregulation in the VSV‐infected group compared with the Mock group was calculated.JCell nucleus and cytoplasm were separated from untreated (Mock) and VSV‐infected iBMMs, RNA was extracted for RT–qPCR analysis and compared with Mock group. Data information: Data of (A‐J) are shown as the mean ± s.d. from three independent experiments. *P < 0.05 and **P < 0.01 by unpaired Student’s *t*‐test.

**Figure EV1:**
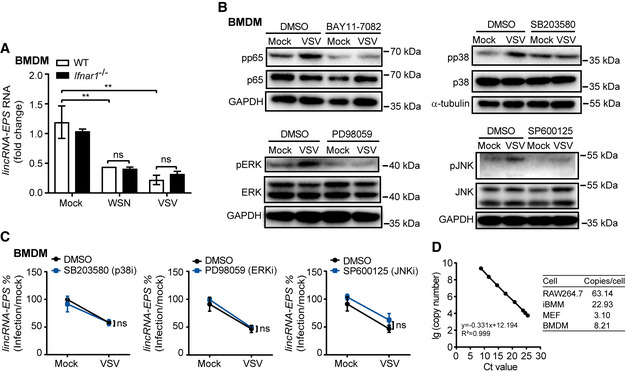
The signaling pathways modulating lincRNA‐EPS expression RT–qPCR analysis of lincRNA‐EPS transcripts in the WT and *Ifnar1* ^−/−^ BMDMs infected with WSN (MOI 1) and VSV (MOI 1) for 6 h.BMDMs were pretreated with NF‐κB inhibitor BAY11‐7082 (1 μM), p38 MAPK inhibitor SB203580 (5 μM), ERK inhibitor PD98059 (50 μM) and JNK inhibitor SP600125 (20 μM) for 1 h prior and infected with VSV (MOI 1) for 6 h. The phosphorylation and total proteins were detected by Western blot. GAPDH and α‐tubulin were shown as loading control.The percentage of lincRNA‐EPS transcripts downregulated in the VSV‐infected group compared with Mock group were calculated.Copy‐number analysis of lincRNA‐EPS transcripts in several cell types by RT–qPCR. Standard curve was generated using *in vitro* transcribed RNA molecule of lincRNA‐EPS as template. Data information: Data of (A, C) are shown as the mean ± s.d. from three independent experiments, **P < 0.01 and ns, not significant by unpaired Student’s *t*‐test. Data of (B, D) are representative results from three independent experiments.

To check the impact of viral infection on lincRNA‐EPS expression, mouse bone marrow‐derived macrophages (BMDMs) were infected with several viruses including RNA viruses VSV and SeV, and DNA virus HSV‐1. All three viruses dramatically suppressed the expression of lincRNA‐EPS (Fig 1A). Transfection of viral RNA mimics polyI:C and viral DNA mimics polydA:dT also led to the downregulation of lincRNA‐EPS expression in BMDMs (Fig 1B). To further confirm whether host antiviral immunity regulates lincRNA‐EPS expression, BMDMs were treated with IFN‐α and IFN‐β, the key cytokines which are always induced during viral infection. Both IFN‐Is significantly suppressed lincRNA‐EPS expression at the very early stage (2 h post stimulation) and at the concentration as low as 20 U/ml (Fig 1C–F). Consistently, higher expression of lincRNA‐EPS was detected in the *Ifnar1* ^−/−^ BMDMs than the WT BMDMs during cells were transfected with polyI:C or infected with SeV (Fig 1G and H). However, lincRNA‐EPS expression was still downregulated in the polyI:C‐transfected and SeV‐infected *Ifnar1* ^−/−^ BMDMs (Fig 1G and H). In addition, there were no differences of lincRNA‐EPS expression between *Ifnar1* ^−/−^ and WT BMDMs when the cells were infected with WSN and VSV (Fig EV1A), which suggest an IFN‐I‐independent pathway also contributed to suppress lincRNA‐EPS expression during viral infection. Hence, we used inhibitors to specifically target NF‐κB, p38, ERK, and JNK signaling pathways (Fig EV1B), which are also activated during viral infection. Inhibition of NF‐κB rather than the MAPK pathways significantly reversed the downregulation of lincRNA‐EPS triggered by VSV infection (Figs 1I and EV1C).

We further checked the cellular localization and abundance of lincRNA‐EPS in macrophages. Similar to the lncRNA Neat1 which mainly localizes in the nucleus (Clemson *et al*, 2009), about 71% of lincRNA‐EPS were detected in the nucleus. A more robust reduction of cytoplasmic lincRNA‐EPS was observed than the nuclear lincRNA‐EPS, although both nuclear and cytoplasmic lincRNA‐EPS were significantly suppressed during VSV infection (Fig 1J). About 63, 23, and 8 copies per cell of lincRNA‐EPS were detected in the RAW264.7, immortalized BMDMs (iBMMs), and BMDMs, respectively (Fig EV1D), indicating higher abundance of lincRNA‐EPS in the macrophage cell lines than the primary macrophages.

Taken together, lincRNA‐EPS was downregulated during host immunity against viral infection, in IFN‐I‐ and NF‐κB‐dependent manners.

### LincRNA‐EPS facilitates viral infection in macrophages

**Figure 2:**
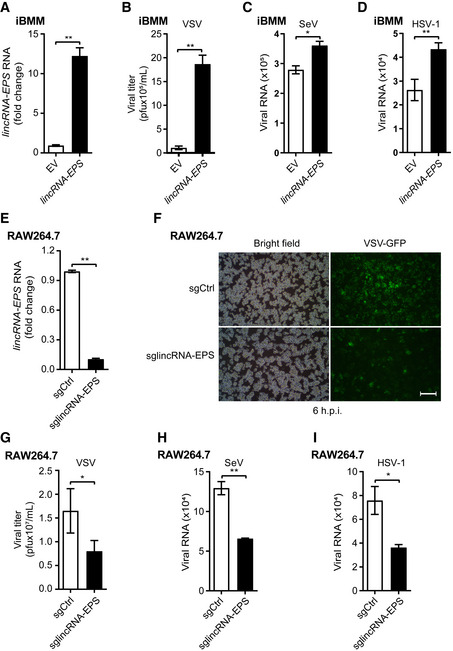
LincRNA‐EPS facilitates viral infection in macrophages ART–qPCR analysis of lincRNA‐EPS transcripts in the lincRNA‐EPS‐stably‐overexpressed iBMMs (*lincRNA‐EPS*) and corresponding control cells (EV).B
*LincRNA‐EPS* iBMMs were infected with VSV (MOI 1) for 8 h, VSV titers were measured by plaque assay.C, DThe EV and *lincRNA‐EPS* iBMMs were infected with SeV (MOI 1) (C) or HSV‐1 (MOI 5) (D) for 12 h. The viral RNA was measured by RT–qPCR.EThe transcripts level of *lincRNA‐EPS* in control (sgCtrl) and lincRNA‐EPS knockdown (sglincRNA‐EPS) RAW264.7 cells were measured by RT–qPCR.F, GsgCtrl and sglincRNA‐EPS RAW264.7 cells were infected with VSV‐GFP (MOI 0.1) for 6 h or infected with VSV (MOI 0.1) for 8 h. The fluorescence of GFP was checked by microscope (F) and the viral titer of VSV from the cell supernatant was measured by plaque assay (G).H, IsgCtrl and sglincRNA‐EPS RAW264.7 cells were infected with SeV (MOI 1) for 8 h (H) and HSV‐1 (MOI 5) for 12 h (I). The viral RNA was measured by RT–qPCR. Data information: Data of (A–E) and (G–I) are shown as the mean ± s.d. from three independent experiments. *P < 0.05 and **P < 0.01 by unpaired Student’s *t*‐test. Data of (F) are representative images from three independent experiments, scale bar, 100 μm.

Next, we sought to investigate the function of lincRNA‐EPS during host innate immunity against viral infection by using macrophage cell lines. We stably overexpressed lincRNA‐EPS in iBMMs (Fig 2A). More VSV, SeV, and HSV‐1 infections were detected in the lincRNA‐EPS‐overexpressed iBMMs than the control cells (Fig 2B–D), which suggested that lincRNA‐EPS broadly facilitated viral infection. Meanwhile, we used a pair of sgRNAs to efficiently knock down the lincRNA‐EPS expression in RAW264.7 cells, a macrophage cell line that is susceptible to multiple viruses and expresses high level lincRNA‐EPS (Fig 2E). Less VSV‐GFP‐infected cells were observed in the lincRNA‐EPS knockdown cells than the control cells (Fig 2F). Consistently, less VSV titer, fewer SeV and HSV‐1 viral genes were detected in the lincRNA‐EPS knockdown cells than the control cells (Fig 2G–I). These results indicated that lincRNA‐EPS facilitated viral infection in macrophages.

### Knockout of lincRNA‐EPS enhances host antiviral ability

**Figure 3:**
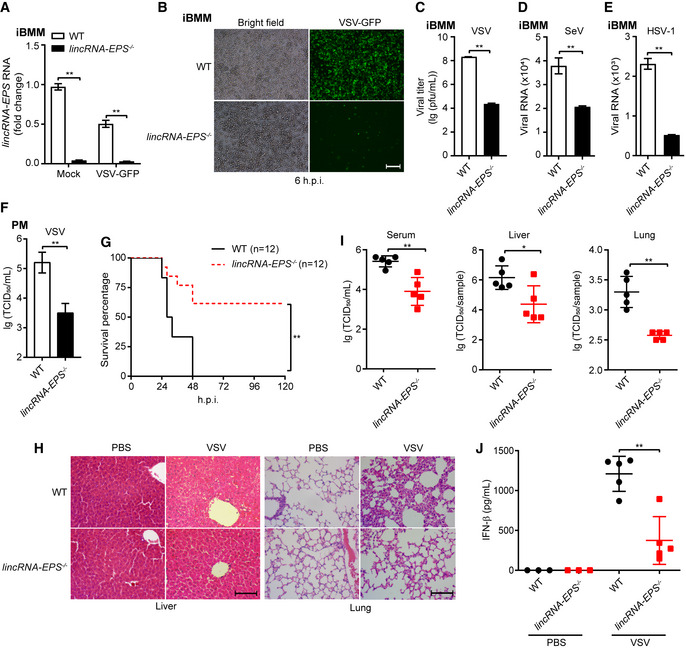
Knockout of lincRNA‐EPS enhances host antiviral ability A, BWT and *lincRNA‐EPS* ^−/−^ iBMMs were infected with VSV‐GFP (MOI 0.1) for 6 h, and the lincRNA‐EPS transcripts were measured by RT–qPCR (A), and the fluorescence of GFP were checked by microscope (B).CCell supernatant from VSV‐infected WT and *lincRNA‐EPS* ^−/−^ iBMMs (MOI 0.1, 8 h) were collected and the viral titer were measured by plaque assay.D, EWT and *lincRNA‐EPS* ^−/−^ iBMMs were infected with SeV (MOI 1) for 8 h (D) or HSV‐1 (MOI 5) for 12 h (E). The viral RNA was measured by RT–qPCR.FPeritoneal macrophages isolated from WT and *lincRNA‐EPS* ^−/−^ mice were infected with VSV (MOI 1) for 10 h, and the viral titer were measured by TCID_50_ assay.GEight weeks female *lincRNA‐EPS* ^−/−^ mice (*n* = 12) and WT littermates (*n* = 12) were injected (*i.v*.) with VSV (lethal dose, 1 × 10^8^ pfu/g), and the survival situation was monitored for 120 h.H–JEight weeks female *lincRNA‐EPS* ^−/−^ mice (*n* = 5) and WT littermates (*n* = 5) were injected (*i.v*.) with VSV (sub‐lethal dose, 6 × 10^7^ pfu/g) for 12 h, and negative control groups were injected with PBS (*n* = 3). Pathological section of liver and lung were harvested by H&E staining (H). Serum, liver, and lung from the VSV‐infected mice were collected. The viral load of serum and tissue homogenate were measured by TCID_50_ assay (I), and the serum IFN‐β protein level were checked by ELISA (J). Data information: Data of (A, C–F) are shown as the mean ± s.d. from three independent experiments, data of (I, J) are shown as the mean ± s.d. of a typical representative result from three independent experiments, and one dot represents a mouse, *P < 0.05 and **P < 0.01 by unpaired Student’s *t*‐test. Data of (G) are calculated with Log‐rank (Mantel‐Cox) test, **P < 0.01. Data of (B, H) are representative images from at least three independent experiments, scale bar,100 μm.

**Figure EV2:**
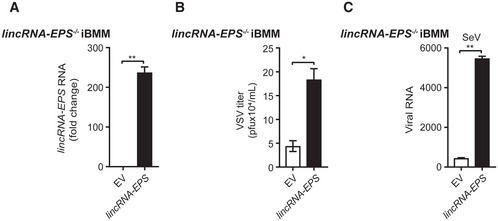
Rescued expression of lincRNA‐EPS inhibits host antiviral ability AThe lincRNA‐EPS transcripts were measured by RT–qPCR in the control (EV) and lincRNA‐EPS‐rescued (*lincRNA‐EPS*) iBMMs.B, CLincRNA‐EPS‐rescued iBMMs and control group were infected with VSV (MOI 1) and SeV (MOI 1) for 12 h, VSV titers were measured by plaque assay (B) and SeV RNA level were checked by RT–qPCR (C). Data information: All data are shown as the mean ± s.d. from three independent experiments, *P < 0.05 and **P < 0.01 by unpaired Student’s *t*‐test.

To further confirm the function of lincRNA‐EPS in facilitating viral infection, we immortalized the *lincRNA‐EPS* ^−/−^ and WT BMDMs (Fig 3A). Much less GFP‐positive cells were observed in the VSV‐GFP‐infected *lincRNA‐EPS* ^−/−^ iBMMs than the WT cells (Fig 3B). Plaque assay results also showed that *lincRNA‐EPS* ^−/−^ iBMMs were more resistant to VSV than the WT iBMMs (Fig 3C). Similarly, as the phenotypes observed in the lincRNA‐EPS knockdown RAW264.7 cells, less SeV and HSV‐1 viral genes were detected in the *lincRNA‐EPS* ^−/−^ iBMMs than the WT cells (Fig 3D and E). However, rescued expression of lincRNA‐EPS in the *lincRNA‐EPS* ^−/−^ iBMMs facilitated VSV and SeV infection (Fig EV2A–C), which indicated that knockout of *lincRNA‐EPS* rather than the off‐target effects of the sgRNAs regulated the host susceptibility to viral infection. Next, we isolated the primary peritoneal macrophages (PMs) to further validate the function of lincRNA‐EPS. *LincRNA‐EPS* ^−/−^ PMs showed more resistant to the VSV than the WT cells (Fig 3F). In addition to the *in vitro* experiments, we further challenged the WT and *lincRNA‐EPS* ^−/−^ mice with VSV to investigate the function of lincRNA‐EPS *in vivo*. The *lincRNA‐EPS* ^−/−^ mice exhibited a much higher survival rate than the WT group during the mice infected with a lethal dose of VSV intravenously (Fig 3G). Consistently, alleviated liver and lung injuries including fewer inflammatory cells infiltration, less liver fibrotic septa, less alveolar wall thickening, and less alveolar cavity atrophy were observed in the *lincRNA‐EPS* ^−/−^ mice than the WT group during the mice infected with a sublethal dose of VSV (Fig 3H). Moreover, less viral load in serum, livers, and lungs was detected in the VSV‐infected *lincRNA‐EPS* ^−/−^ mice comparing to the WT mice (Fig 3I). Interestingly, lower serum IFN‐β was detected in the VSV‐infected *lincRNA‐EPS* ^−/−^ mice than in the WT group (Fig 3J). Together, these results demonstrated that lincRNA‐EPS facilitated viral infection such as VSV infection *in vitro* and *in vivo*, likely in an IFN‐I‐independent manner.

### Greater induction of antiviral ISGs in the *lincRNA‐EPS* ^−/−^ macrophages

**Figure 4:**
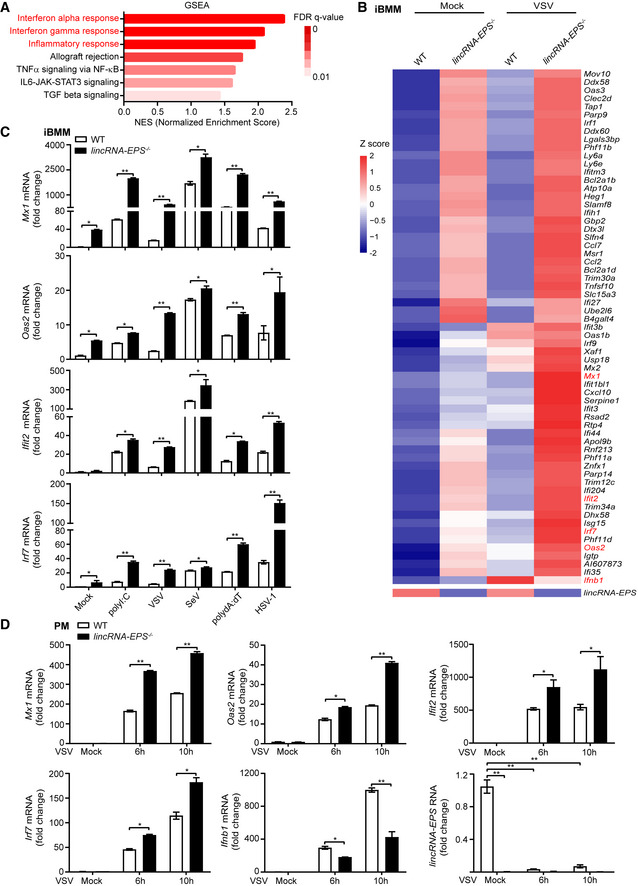
Greater induction of antiviral ISGs in the *lincRNA‐EPS* ^−/−^ macrophages Overview of GSEA analysis using the whole transcriptome of RNA‐seq from biological duplicates of VSV (MOI 0.1)‐infected WT and *lincRNA‐EPS* ^−/−^ iBMMs for 6 h.The mean RPKM values of the biological duplicates of Mock or VSV‐infected WT and *lincRNA‐EPS* ^−/−^ iBMMs were calculated, and the upregulated antiviral genes of *lincRNA‐EPS* ^−/−^ iBMMs comparing to WT iBMMs were listed by heatmap.RT–qPCR analysis of *Mx1*, *Oas2, Ifit2, Irf7* mRNA level in the WT and *lincRNA‐EPS* ^−/−^ iBMMs transfected with 1 μg/ml polyI:C, polydA:dT or infected with VSV (MOI 0.1) and SeV (MOI 1) for 6 h, or HSV (MOI 5) for 12 h.RT–qPCR analysis of *Mx1*, *Oas2, Ifit2, Irf7, Ifnb1* mRNA level and lincRNA‐EPS transcripts in the WT and *lincRNA‐EPS* ^−/−^ PMs infected with VSV (MOI 1) for 6 h and 10 h. Data information: Data of (C, D) are shown as the mean ± s.d. from three independent experiments, *P < 0.05 and **P < 0.01 by unpaired Student’s *t*‐test.

**Figure EV3:**
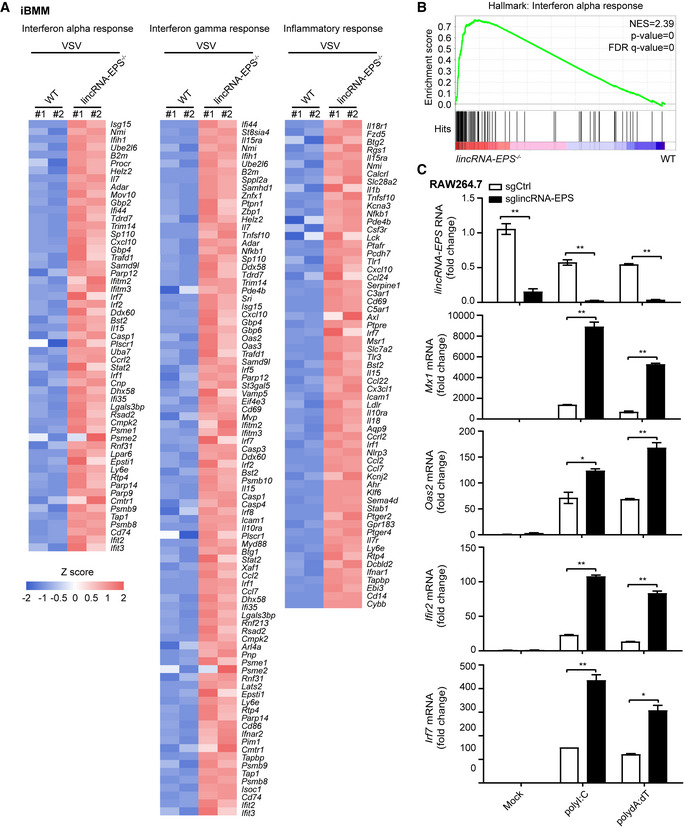
Greater induction of antiviral ISGs in the *lincRNA‐EPS* ^−/−^ macrophages Heatmap analysis of differential genes expression in top three pathways of GSEA.Enrichment plot of Interferon alpha response was extracted from GSEA.Control (sgCtrl) and lincRNA‐EPS knockdown (sglincRNA‐EPS) RAW264.7 cells were transfected with 1 μg/ml polyI:C and polydA:dT for 10 h, the transcripts of *lincRNA‐EPS* and *Mx1*, *Oas2*, *Ifit2*, and *Irf7* were measured by RT–qPCR. Data information: Data of (C) are shown as the mean ± s.d. from three independent experiments, *P < 0.05 and **P < 0.01 by unpaired Student’s *t*‐test.

**Figure EV4:**
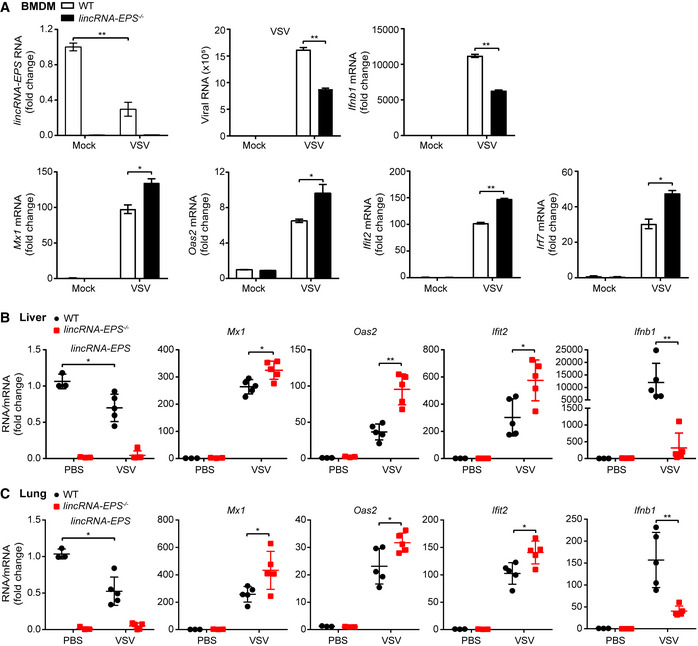
Deficiency of lincRNA‐EPS enhanced ISGs expression in primary macrophages and infected mice model ART–qPCR analysis of *lincRNA‐EPS* transcripts, viral RNA and *Ifnb1, Mx1*, *Oas2, Ifit2, Irf7* mRNA level in the WT and *lincRNA‐EPS* ^−/−^ BMDMs infected with VSV (MOI 5) for 10 h.B, CEight weeks female *lincRNA‐EPS* ^−/−^ mice (*n* = 5) and WT littermates (*n* = 5) were injected (*i.v*.) with VSV (sub‐lethal dose, 6 × 10^7^ pfu/g) for 12 h, and negative control groups were injected with PBS (*n* = 3). RT–qPCR analysis of *lincRNA‐EPS* transcripts and *Mx1*, *Oas2, Ifit2, Ifnb1* mRNA level from liver (B) and lung (C) tissue homogenate. Data information: Data of (A) are shown as the mean ± s.d. from three independent experiments, data of (B, C) are shown as the mean ± s.d. of a typical representative result from three independent experiments, and one dot represents a mouse. *P < 0.05 and **P < 0.01 by unpaired Student’s *t*‐test.

To determine how lincRNA‐EPS facilitates viral infection, we performed RNA sequencing and analyzed the transcriptomes of uninfected and VSV‐infected iBMMs. Gene set enrichment analysis (GSEA) results showed that the interferon alpha response, the interferon gamma response, and the inflammatory response pathways were mostly affected by lincRNA‐EPS deficiency (Figs 4A and EV3A). The majority of these genes in the IFN‐I response gene sets were upregulated in the *lincRNA‐EPS* ^−/−^ iBMMs (Fig EV3B). We selected the upregulated differentially expressed genes (DEGs) in the *lincRNA‐EPS* ^−/−^ iBMMs and overlapped with the ISGs list (Hubel *et al*, 2019). Sixty‐two ISGs were upregulated in the *lincRNA‐EPS* ^−/−^ iBMMs by comparing to the WT cells (Fig 4B). Most of these overlapped ISGs are antiviral genes (Fig 4B). We verified several key antiviral ISGs by RT–qPCR, and found that more induction of *Mx1*, *Oas2, Ifit2*, and *Irf7* were observed in the *lincRNA‐EPS* ^−/−^ iBMMs than the WT cells when the cells infected with VSV, SeV, HSV‐1, or transfected with viral RNA mimics polyI:C or viral DNA mimics polydA:dT (Fig 4C). Basal expressions of multiple antiviral ISGs were also higher in the *lincRNA‐EPS* ^−/−^ iBMMs than the WT iBMMs (Fig 4B and C). In addition, *Mx1*, *Oas2, Ifit2*, and *Irf7* genes were also higher elevated in the VSV‐infected *lincRNA‐EPS* ^−/−^ PMs and the viral mimics‐transfected *lincRNA‐EPS* knockdown RAW264.7 cells than their respective control cells (Figs 4D and EV3C). However, fewer *Ifnb1* transcripts were detected in the VSV‐infected iBMMs and primary PMs than their respective control cells (Fig 4B and D). Moreover, reduced VSV infection, enhanced ISGs expression, and repressed *Ifnb1* transcription were observed in the VSV‐infected *lincRNA‐EPS* ^−/−^ BMDMs (Fig EV4A), and in the liver and lung tissues from VSV‐infected mice (Fig EV4B and C). These results suggested that lincRNA‐EPS suppressed the induction of antiviral ISGs during viral infection *in vitro* and *in vivo*, and thus facilitated viruses such as VSV, SeV, and HSV‐1 infection.

### Stronger activation of IFNAR downstream signaling independent of IFN‐β induction in the *lincRNA‐EPS* ^−/−^ macrophages

**Figure 5:**
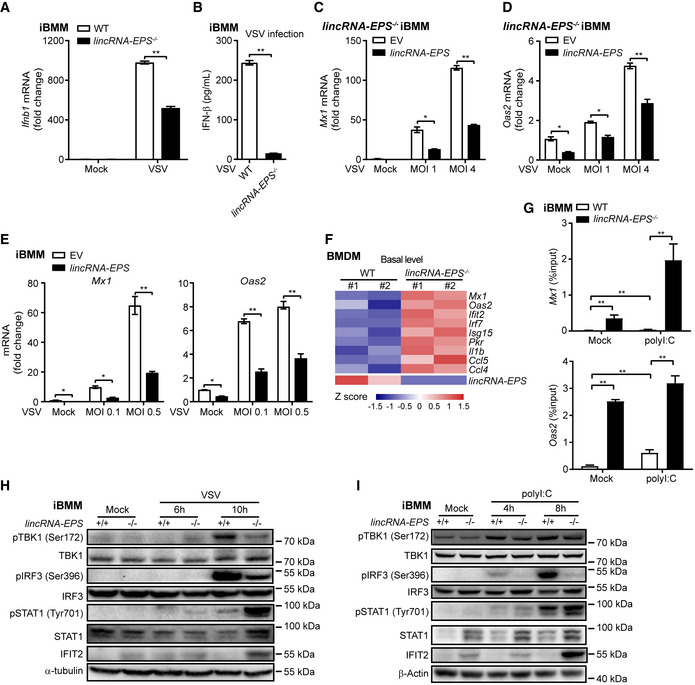
Stronger activation of IFNAR downstream signaling independent of IFN‐β induction in the *lincRNA‐EPS* ^−/−^ macrophages A, BRT–qPCR analysis of *Ifnb1* mRNA level in the WT and *lincRNA‐EPS* ^−/−^ iBMMs infected with VSV (MOI 0.1) for 6 h (A), ELISA analysis of supernatant IFN‐β protein after infecting for 8 h (B).C, D
*Mx1* (C) and *Oas2* (D) mRNA level were measured in the control (EV) and lincRNA‐EPS‐rescued (*lincRNA‐EPS*) iBMMs after infecting with VSV with different titer for 6 h.ELincRNA‐EPS‐stably overexpressed iBMMs (*lincRNA‐EPS*) and corresponding control group (EV) were infected with VSV for different titer (MOI 0.1 and MOI 0.5), *Mx1* and *Oas2* mRNA level were measured by RT–qPCR after 6 h.FThe basal level of ISGs and proinflammatory genes in the WT and *lincRNA‐EPS* ^−/−^ BMDMs (Datasets from the ArrayExpress database under the accession number E‐MTAB‐4088) were listed by heatmap.GChIP assays using anti‐H3K4me3 antibody was carried out in WT and *lincRNA‐EPS* ^−/−^ iBMMs with or without polyI:C (1 μg/ml) transfection for 4 h, *Mx1* and *Oas2* enrichment relative to input were measured by qPCR.H, IWT and *lincRNA‐EPS* ^−/−^ iBMMs were infected with VSV (MOI 0.1) for 6 or 10 h (H) or transfected with polyI:C (1 μg/ml) for 4 or 8 h (I), phosphorylation and total protein expression were analyzed by Western blot with α‐tubulin and β‐Actin as loading controls. Data information: data of (A–E) and (G) are shown as the mean ± s.d. from three independent experiments, *P < 0.05 and **P < 0.01 by unpaired Student’s *t*‐test. Data of (H, I) are representative results from three independent experiments.

**Figure EV5:**
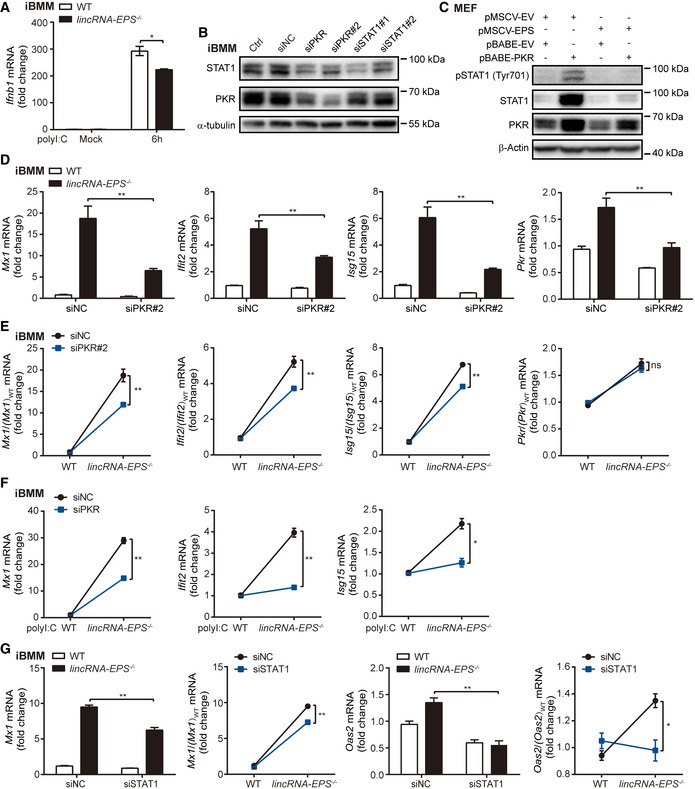
LincRNA‐EPS inhibits PKR‐STAT1‐dependent induction of ISGs ART–qPCR analysis of *Ifnb1* mRNA level of polyI:C (1 μg/ml) transfected iBMMs for 6 h.B20 nM siRNA for negative control (NC), PKR and STAT1 were transfected into iBMMs for 48 h. STAT1 and PKR protein level relative to naïve cells (Ctrl) were detected by Western blot and α‐tubulin was shown as a loading control.CEmpty vector (pMSCV‐EV) and pMSCV‐PIG‐lincRNA‐EPS (pMSCV‐EPS) were transfected into PKR‐overexpressed MEF cell lines (pBABE‐PKR) and control cells (pBABE‐EV), phosphorylation and total levels of STAT1 and PKR were detected by Western blot and β‐Actin was shown as a loading control.D, E20 nM siRNA for NC (siNC) and PKR (siPKR#2) were transfected into WT and *lincRNA‐EPS* ^−/−^ iBMMs, *Mx1, Ifit2, Isg15 and Pkr* mRNA level were measured by RT–qPCR (D), and relative basal expression change rates of the antiviral ISGs in *lincRNA‐EPS* ^−/−^ iBMMs were calculated compared with WT group (E).F20 nM siRNA for NC and PKR were transfected into WT and *lincRNA‐EPS* ^−/−^ iBMMs following transfected with 1 μg/ml polyI:C for 6 h, *Mx1, Ifit2, Isg15* mRNA level were measured by RT–qPCR. Relative expression change rates of ISGs in *lincRNA‐EPS* ^−/−^ iBMMs were calculated compared with WT group.G20 nM siRNA for NC and STAT1 were transfected into WT and *lincRNA‐EPS* ^−/−^ iBMMs, *Mx1, Oas2* mRNA level were measured by RT–qPCR. Relative expression change rates of ISGs in *lincRNA‐EPS* ^−/−^ iBMMs were calculated compared with WT group. Data information: Data of (A, and D–G) are shown as the mean ± s.d. from three independent experiments, *P < 0.05 and **P < 0.01 by unpaired Student’s *t*‐test. Data of (B–C) are representative images from three independent experiments.

Induction of IFN‐I is essential for the host against acute viral infection (McNab *et al*, 2015). However, VSV infection‐induced serum IFN‐β and *Ifnb1* transcripts were downregulated in the *lincRNA‐EPS* ^−/−^ mice and primary macrophages (Figs 3J, 4D, EV4A–C). In the VSV‐infected iBMMs, we also confirmed that fewer *Ifnb1* transcripts and supernatant IFN‐β proteins were detected in the *lincRNA‐EPS* ^−/−^ cells than the WT cells (Fig 5A and B). To eliminate the influence of inhibited viral infection by deficiency of lincRNA‐EPS on IFN‐β upstream signaling, we activated iBMMs by transfecting polyI:C instead of VSV infection. Less *Ifnb1* transcripts were detected in the *lincRNA‐EPS* ^−/−^ iBMMs than the WT cells (Fig EV5A). Rescued expression of lincRNA‐EPS in the *lincRNA‐EPS* ^−/−^ iBMMs or overexpression of lincRNA‐EPS in WT iBMMs significantly inhibited the induction of antiviral genes including *Mx1* and *Oas2* (Fig 5C–E), which was consistent with the results that *lincRNA‐EPS* ^−/−^ mice and macrophages exhibited stronger antiviral abilities than their respective controls. Knockout of lincRNA‐EPS upregulated the basal expression level of numerous antiviral genes including *Mx1*, *Oas2, Ifit2, and Irf7* in BMDMs (Fig 5F). The histone modification, trimethylation of lysine 4 in histone H3 (H3K4me3) that localized to the 5′ regions of target genes, is associated with high transcription activity (Liang *et al*, 2004). Thus, we checked the H3K4me3 modification in the promoters of several antiviral ISGs by ChIP‐qPCR. Transfection of polyI:C dramatically induced the H3K4me3 modifications in the promoters of *Mx1* and *Oas2* in both WT and *lincRNA‐EPS* ^−/−^ iBMMs, while knockout of lincRNA‐EPS with or without polyI:C transfection induced higher level of H3K4me3 modifications in the promoters of these genes (Fig 5G). Moreover, upon VSV infection, activation of IFN‐β upstream signaling including phosphorylation of TBK1 and IRF3 was attenuated while IFN‐β downstream activation including STAT1 phosphorylation was elevated in the *lincRNA‐EPS* ^−/−^ iBMMs, which was further verified in the polyI:C‐transfected macrophages to eliminate the indirect effect of inhibited viral load on the IFN‐β upstream signaling (Fig 5H and I).

Taken together, these results demonstrated that knockout of lincRNA‐EPS suppressed IFN‐I upstream signaling activation and IFN‐β induction. However, IFNAR downstream signaling activation and antiviral ISGs were highly induced in the *lincRNA‐EPS* ^−/−^ macrophages.

### LincRNA‐EPS and viral RNA competitively interact with PKR

**Figure 6:**
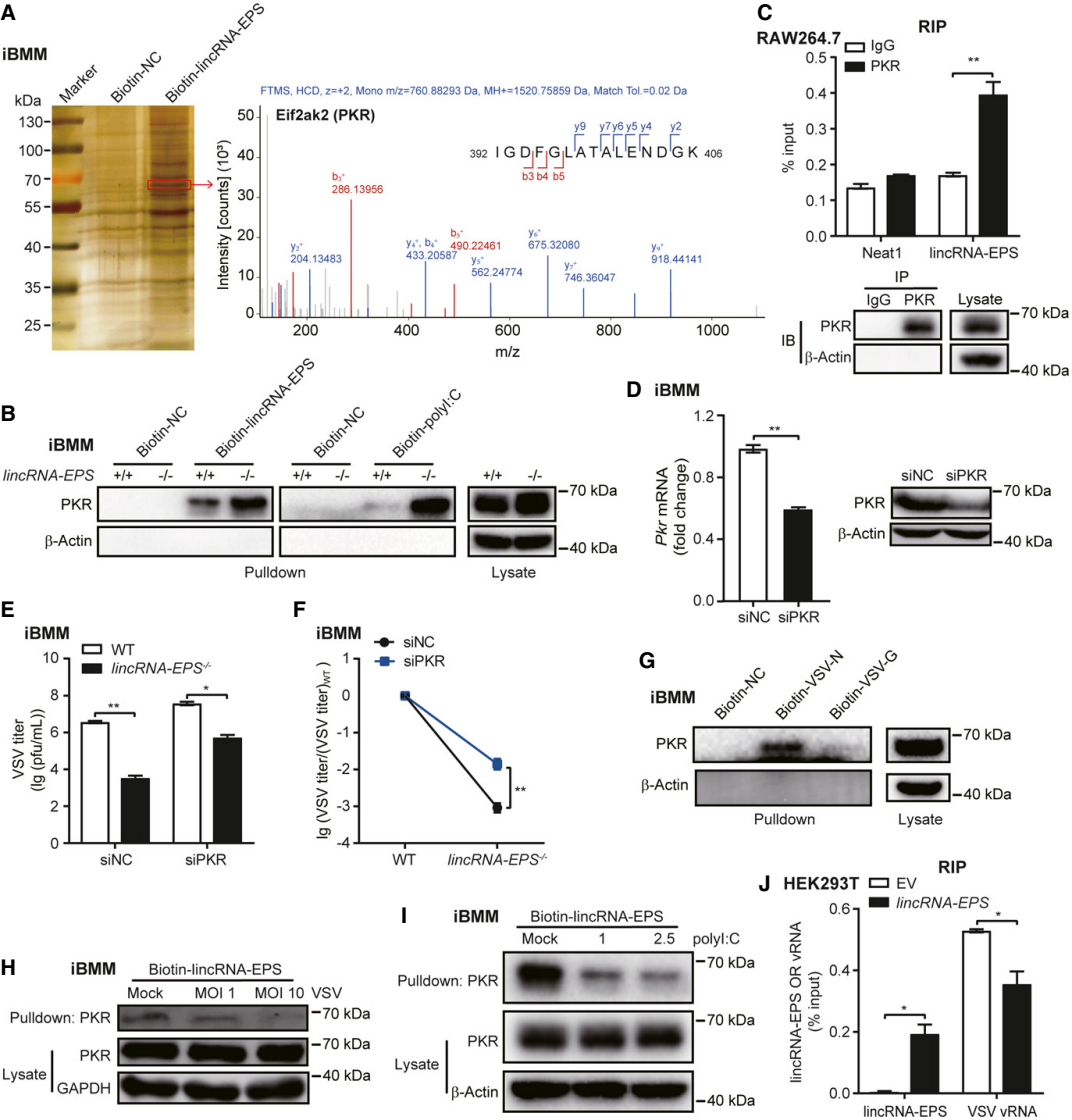
LincRNA‐EPS and viral RNA competitively interact with PKR ALincRNA‐EPS and negative control RNA (NC) were transcribed *in vitro* to label desthiobiotin and pulldown proteins from iBMMs lysate, the differential protein bands from silver‐stained SDS‐PAGE were cut for mass spectrometry analysis. One of the identified proteins from the band in the red box was PKR.BWestern blot for verification of RNA pulldown‐MS results with β‐Actin as a negative loading control.CThe interaction between PKR and lincRNA‐EPS were checked by RIP–qPCR using anti‐PKR antibody, and the efficiency of IP was verified by Western blot.D20 nM siRNA targeting negative control (NC) and PKR were transfected into iBMMs for 48 h, the mRNA and protein level of PKR were tested by RT–qPCR and Western blot, respectively.E, FVSV titer in the WT and *lincRNA‐EPS* ^−/−^ iBMMs transfected with siRNA as described in (D) and infected with VSV (MOI 0.1) for 8 h was measured by plaque assay (E). The alteration rates of viral titers were calculated in *lincRNA‐EPS* ^−/−^ iBMMs relative to WT group (F).GVSV‐N and VSV‐G mRNA were transcribed from virus particles for RNA‐pulldown, the interactions between VSV RNA with PKR protein was checked by Western blot, β‐Actin was shown as a negative control.H
*LincRNA‐EPS* was transcribed *in vitro* and pulled down proteins from iBMMs infected with VSV for 4 h, PKR protein was detected by Western blot and GAPDH was shown as a loading control.IRNA‐pulldown using biotinylated lincRNA‐EPS from cell lysates of polyI:C (1 and 2.5 μg/ml) transfected iBMMs for 4 h, PKR protein was detected by Western blot and β‐Actin was shown as a loading control.JHEK293T cells transfected with lincRNA‐EPS and Flag‐tagged PKR were infected with VSV to detect the PKR‐binding *lincRNA‐EPS* and VSV RNA by RIP–qPCR using anti‐Flag antibody. Data information: Data of (C, up panel), (D, left panel), (E, F) and (J) are shown as the mean ± s.d. from three independent experiments, *P < 0.05 and **P < 0.01 by unpaired student *t*‐test. Data of (B), (C, down panel), (D, right panel), (G, H, I) are representative images from three independent experiments.

LincRNA‐EPS tends to function through recruiting the RNA‐binding protein hnRNPL in the nucleus to restrain chromatin accessibility at the promoters of interferon regulated genes in macrophages (Atianand *et al*, 2016; Agliano *et al*, 2019). To study how lincRNA‐EPS inhibits IFNAR downstream signaling and thus restricts host antiviral immunity, we checked all the lincRNA‐EPS‐interacted proteins in the whole cell lysates of macrophages by RNA pulldown and mass spectrometry technology. Among the numerous candidates, lincRNA‐EPS strongly interacted with the dsRNA‐binding protein PKR and the positive control protein hnRNPL (Fig 6A, Table EV1). The interaction between PKR and lincRNA‐EPS was confirmed by using biotinylated lincRNA‐EPS and polyI:C RNA pulldown assay (Fig 6B). Next, we validated the native interaction between PKR and lincRNA‐EPS in macrophages by pulling down the endogenous PKR proteins to analyze the associated RNA molecules by RT–qPCR (Fig 6C). To further explore whether PKR is involved in the lincRNA‐EPS‐mediated regulation of antiviral immunity, we knocked down PKR by RNAi technology in both WT and *lincRNA‐EPS* ^−/−^ iBMMs. Once the expression of PKR was efficiently downregulated (Figs 6D and EV5B), the reduction of VSV infection in the *lincRNA‐EPS* ^−/−^ iBMMs was significantly reversed, although knockout of lincRNA‐EPS still enhanced the host antiviral immunity against invaded VSV in both siNC and siPKR iBMMs (Fig 6E and F).

PKR was well‐known as a viral RNA sensor to recognize virus‐derived RNA molecules including VSV genomic RNA (Stojdl *et al*, 2000; Dauber & Wolff, 2009). VSV transcripts include the leader region at the 3′ genomic promoter, N‐P‐M‐G‐L encoding viral genes, and the 5′ trailer region (Villarreal *et al*, 1976). The VSV viral RNA fragment of leader/N junction is able to bind and trigger RNA sensor RIG‐I (Linder *et al*, 2021). We used VSV RNA fragments to pulldown PKR and found that VSV‐N but not VSV‐G strongly interacted with PKR (Fig 6G). To investigate whether VSV viral RNA and lincRNA‐EPS competitively bind with PKR, we activated iBMMs with different doses of VSV or polyI:C, and extracted cell lysates for lincRNA‐EPS RNA‐pulldown assay. The Western blot results indicated that VSV infection and polyI:C transfection hindered the interaction between lincRNA‐EPS and PKR in a dose‐dependent manner (Fig 6H and I). The RIP‐RT–qPCR assay for testing the ability of PKR in binding with lincRNA‐EPS and VSV viral RNA demonstrated that overexpression of lincRNA‐EPS could antagonize the interaction between PKR and VSV viral RNA (Fig 6J), which further confirmed that lincRNA‐EPS inhibited antiviral immunity through restraining PKR sensing viral RNA.

### LincRNA‐EPS inhibits PKR‐STAT1‐dependent induction of ISGs

**Figure 7:**
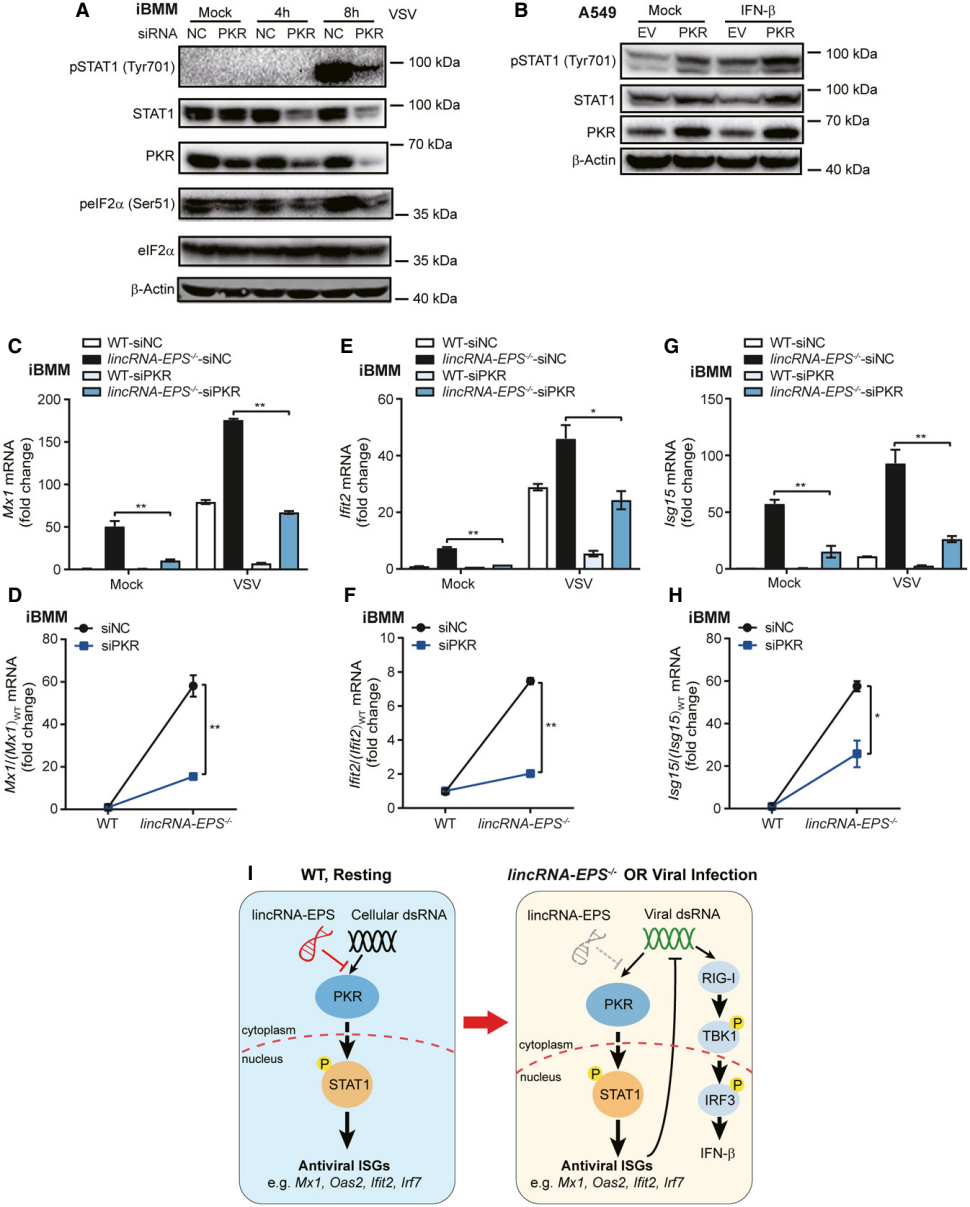
LincRNA‐EPS inhibits PKR‐STAT1‐dependent induction of ISGs A20 nM siRNA for negative control (NC) and PKR were transfected into iBMMs following infected with VSV (MOI 0.1) for 4 h or 8 h, phosphorylation and total levels of STAT1, eIF2α and PKR were detected by Western blot and β‐Actin was shown as a loading control.BPKR was transfected into A549 cells for 24 h following stimulated with IFN‐β (50 ng/ml) for 1 h to test STAT1 phosphorylation by Western blot and β‐Actin was shown as a loading control.C–H20 nM siRNA for NC and PKR were transfected into WT and *lincRNA‐EPS* ^−/−^ iBMMs following infection with VSV (MOI 0.1) for 4 h, *Mx1, Ifit2,* and *Isg15* mRNA level were measured by RT–qPCR (C, E, and G), and relative basal expression change rates of the antiviral ISGs in *lincRNA‐EPS* ^−/−^ iBMMs were calculated compared with WT group (D, F, and H).IWorking model of lincRNA‐EPS in resting and viral infected WT or *lincRNA‐EPS* ^−/−^ cells. Data information: Data of (A, B) are representative images from three independent experiments. Data of (C‐H) are shown as the mean ± s.d. from three independent experiments, *P < 0.05 and **P < 0.01 by unpaired student *t*‐test.

PKR is required for phosphorylation and activation of STAT1 under the response to IFN‐γ and LPS (Ramana *et al*, 2000; Lee *et al*, 2005; Karehed *et al*, 2007). During VSV infection, phosphorylation of STAT1 was also attenuated in the PKR knockdown iBMMs (Fig 7A). Overexpression of PKR upregulated the phosphorylation of STAT1 as the IFN‐β treatment (Fig 7B), which suggested a potential IFN‐I‐independent pathway favoring PKR‐trigged phosphorylation of STAT1. In addition, overexpression of lincRNA‐EPS repressed STAT1 phosphorylation and total STAT1 protein level which were induced by ectopic expressed PKR in MEF cells (Fig EV5C).

To investigate whether lincRNA‐EPS involves in the regulation of PKR‐STAT1 signaling, we checked the basal expression of antiviral ISGs including *Mx1*, *Ifit2*, and *Isg15* after PKR knockdown in iBMMs. Upregulation of *Mx1*, *Ifit2*, and *Isg15* in *lincRNA‐EPS* ^−/−^ iBMMs were attenuated after PKR knockdown (Figs 7C–H and EV5D and E). Consistently, induction of *Mx1*, *Ifit2* and *Isg15* were also attenuated after PKR knockdown in the VSV‐infected and polyI:C‐transfected *lincRNA‐EPS* ^−/−^ iBMMs (Figs 7C and E, and G, and EV5F). Moreover, knockdown of STAT1, the key transcription factor of the PKR‐STAT1 axis, significantly decreased the basal expression of lincRNA‐EPS‐regulated ISGs *Mx1* and *Oas2* (Fig EV5G).

In summary, our study has demonstrated that lincRNA‐EPS negatively regulated PKR‐STAT1‐dependent antiviral immunity. In addition to the inhibitory role of lincRNA‐EPS on restraining chromatin accessibility by associating with hnRNPL in nucleus (Atianand *et al*, 2016), the cytosolic lincRNA‐EPS is able to repress PKR‐STAT1 axis and downregulate ISGs expression in the resting cells. Downregulation of lincRNA‐EPS during viral infection or genetic knockout of lincRNA‐EPS facilitates PKR‐STAT1 signaling axis induced antiviral genes expression, such as *Mx1*, *Oas2*, *Ifit2*, and *Irf7* (Fig 7I). PKR‐mediated STAT1 activation and ISGs induction directly inhibit viral infection, which may result in less RIG‐I‐TBK1‐IRF3 signaling activation and less IFN‐β production.

## Discussion

LncRNAs are widely expressed in immune cells and precisely regulated during innate immunity against viral infection (Ouyang *et al*, 2016; Atianand *et al*, 2017). Here, our study has indicated that downregulation of lincRNA‐EPS in macrophages significantly facilitates host antiviral innate immunity *in vitro* and *in vivo*. LincRNA‐EPS not only acts as a transcriptional brake to restrain PAMP‐ and DAMP‐triggered inflammation (Atianand *et al*, 2016; Chen *et al*, 2021), but also negatively regulates host antiviral immune responses. Decreased expression of lincRNA‐EPS at the early stage of viral infection benefits the host cells by enhancing the ability to clear the invaded viruses. Higher expression of lincRNA‐EPS in the resting macrophages or recovered expression of lincRNA‐EPS after clearance of viruses also helps the host to maintain homeostasis and avoid autoimmunity.

**Figure EV6:**
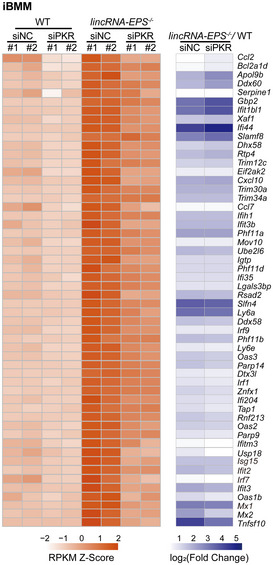
Downregulated ISGs after interfering PKR expression 20 nM siRNAs for negative control (siNC) and PKR (siPKR) were transfected into iBMMs for 48 h, and the total RNA was isolated to perform RNA sequencing. The mean RPKM values of the biological duplicates were calculated and the downregulated antiviral ISGs after knocking down PKR were listed by heatmap (left panel). The fold change of ISGs level of *lincRNA‐EPS* ^−/−^ iBMMs comparing to the WT iBMMs in siNC and siPKR groups were also illustrated by heatmap (right panel).

Knockout of lincRNA‐EPS changes the repressed chromatin state to a more accessible chromatin state at the promoters of selected IRGs including key antiviral genes (Atianand *et al*, 2016). However, it is well accepted that accessibility of promoter DNA is not sufficient to drive gene transcription (Ernst & Kellis, 2013; Chereji *et al*, 2019). Our study describes the cytoplasmic function of lincRNA‐EPS in the regulation of antiviral immunity. Therefore, it is possible that cytoplasmic and nuclear lincRNA‐EPS synergistically suppress ISG expression at both resting and viral infected conditions. Nuclear lincRNA‐EPS‐hnRNPL complex determines chromatin accessibility of the ISG promoters, and the cytoplasmic lincRNA–EPS–PKR–STAT1 signaling axis controls ISG transcription. Transcriptome analysis of resting WT and *lincRNA‐EPS* ^−/−^ macrophages with PKR knockdown did not show any specific genes regulated by lincRNA–EPS–PKR–STAT1 axis comparing to lincRNA‐EPS‐hnRNPL axis (Fig EV6), which suggest that cytoplasmic and nuclear lincRNA–EPS may have synergistical functions in regulating host antiviral immunity.

Multiple lncRNAs have been reported to inhibit viral infection via the IFN‐I‐dependent pathways. Lnczc3h7a associates with TRIM25 and serves as a molecular scaffold to stabilize the interaction between TRIM25 and RIG‐I to strengthen IFN‐I production and antiviral response (Lin *et al*, 2019). LncRNA‐GM enhances IFN‐I production and thus inhibits viral replication by reducing GSTM1‐mediated S‐glutathionylation of TBK1 (Wang *et al*, 2020). LncLrrc55‐AS promotes IRF3 phosphorylation to enhance IFN‐I signaling (Zhou *et al*, 2019). However, there are also numerous lncRNAs that are hijacked by viruses to suppress host immune responses for facilitating viral escape. For instance, LncRNA‐ACOD1 interacts with glutamic‐oxaloacetic transaminase (GOT2) and supports virus infection through modulating cellular metabolic networks (Wang *et al*, 2017a). NRAV, which represses the transcription of ISGs by affecting histone modification, is downregulated once sensing viruses to benefit host antiviral ability (Li *et al*, 2020). LncRNA‐CMPK2 negatively regulates the activation of IFN‐I signaling and induction of ISGs to promote HCV replication *in vitro* and *in vivo* (Kambara *et al*, 2014). Here, we have discovered a new regulator, lincRNA‐EPS, which inhibits antiviral immunity by attenuating the PKR‐STAT1‐dependent induction of antiviral ISGs, while not affecting IFN‐I production. PKR recognizes viral dsRNA from replication or transcription intermediates of a wide range of viruses (Dauber & Wolff, 2009), mtRNAs formed intermolecular dsRNA (Kim *et al*, 2018), cytoplasmic circRNAs formed 16‐26 bp imperfect RNA duplexes (Liu *et al*, 2019), and lncRNA *GRASLND* (Huynh *et al*, 2020). We have found that PKR also interacts with lincRNA‐EPS, which potentially antagonizes the interaction between PKR and viral RNA.

Although there are only 20–30% of lincRNA‐EPS located at the cytoplasm and ~ 23 copies per cell of lincRNA‐EPS in the iBMMs. However, 6 copies of circPOLR2A in Hela cells are sufficient to suppress PKR activation significantly. Reduced circPOLR2A to ~ 1 copy per cell during polyI:C stimulation still inhibits the PKR activation (Liu *et al*, 2019). Therefore, it is possible that 4‐6 copies of cytoplasmic lincRNA‐EPS are sufficient to interact with PKR, block viral RNA, and thus suppress host antiviral immunity.

PKR plays a vital antiviral role through recognizing viral dsRNA, which is derived both from RNA viruses (e.g. VSV and SeV) and DNA viruses (e.g. HSV‐1) (Dauber & Wolff, 2009). Although the classical downstream signaling of PKR is activating eIF2α to suppress viral protein translation and then inhibit virus replication (Dalet *et al*, 2015). However, we did not observe the elevated phosphorylation of eIF2α in the virus‐infected *lincRNA‐EPS* ^−/−^ macrophages. Interestingly, knockdown of PKR by RNAi attenuates STAT1 phosphorylation in the VSV‐infected iBMMs, and overexpression of PKR facilitates STAT1 phosphorylation as IFN‐β treatment in A549 cells, which suggests that PKR is able to activate STAT1 independently of IFN‐I production. Direct interaction between PKR and STAT1 was observed (Wong *et al*, 1997), although it is not thoroughly verified whether this interaction favors phosphorylation of STAT1 and induces STAT1‐targeted antiviral genes. Further studies are required to elucidate the mechanism for PKR‐mediated STAT1 activation after PKR recognizing exogenous or endogenous dsRNA.

RIG‐I is also an important cytoplasmic RNA sensor for most RNA viruses including VSV (Yoneyama *et al*, 2004). RIG‐I‐MAVS‐TBK1‐IRF3‐IFN‐β signaling axis is essential for host antiviral immunity (Honda *et al*, 2006). However, our results show that viral infection or genetic knockout of lincRNA‐EPS facilitates PKR‐STAT1 signaling axis induced antiviral genes such as *Mx1*, *Oas2*, *Ifit2*, and *Irf7*, while inhibits phosphorylation of TBK1 and IRF3, as well as the IFN‐β induction. One explanation is that PKR‐mediated STAT1 activation and ISGs induction directly inhibits viral infection, which may result in less activation of RIG‐I‐TBK1‐IRF3 signaling and less IFN‐β production. Another possibility is that activation of PKR‐STAT1 signaling induces negative regulators of RIG‐I such as lnc‐Lsm3b, an inducible host lncRNA that binds to RIG‐I monomers and inactivates the RIG‐I innate function (Jiang *et al*, 2018).

In the virus‐infected macrophages, we found that the repression of lincRNA‐EPS is not totally dependent on IFN‐I downstream pathway, although both IFN‐α and IFN‐β significantly inhibit lincRNA‐EPS expression. NF‐κB activity is also required for downregulating lincRNA‐EPS during viral infection, which is consistent with the phenotypes observed in the LPS‐stimulated macrophages (Atianand *et al*, 2016; Chen *et al*, 2021). Detail molecular mechanism for dramatic downregulation of lincRNA‐EPS expression within 2 h is unexplored. Whether any transcriptional repressor inhibits lincRNA‐EPS transcriptionally or any RNA‐binding proteins promote lincRNA‐EPS degradation post‐transcriptionally will be investigated in our future studies.

In conclusion, our study has expanded the physiological function of lincRNA‐EPS in host antiviral immunity and certified that host accelerates antiviral capability through restraining expression of lincRNA‐EPS once sensing invaded virus. We have outlined an alternative antiviral pathway that downregulation of lincRNA‐EPS favors PKR‐STAT1‐dependent antiviral ISGs induction.

## Materials and Methods

### Mice

C57BL/6N *lincRNA‐EPS* ^−/−^ mice were generated by Biocytogen (Beijing, China) by using the CRISPR/Cas9 technology as described previously (Chen *et al*, 2021). Briefly, the whole chromosomal region (about 4 kb) of gene *Ttc39aos1* (Gene ID: 102635290) was deleted by a pair of sgRNAs: 5′‐CCGCCCGCTTTCCCGCCTTCTGG‐3′ and 5′‐GCATTACTTGGACAGCCCCTTGG‐3′. Genotyping primers for WT mice were 5′‐TCACTGAATACACAGGCTGCTGCAA‐3′ (lincRNA‐EPS‐F1) and 5′‐GCTTGTACTCGCCTCTTCTCTGCAA‐3′ (lincRNA‐EPS‐R). Genotyping primers for *lincRNA‐EPS* ^−/−^ mice were 5′‐GCAGACAGGCGTGGACATTCATTCT‐3′ (lincRNA‐EPS‐F2) and 5′‐GCTTGTACTCGCCTCTTCTCTGCAA‐3′ (lincRNA‐EPS‐R). PCR products for WT and KO bands were 456 bp and 417 bp, respectively. All the mice were maintained in the specific pathogen‐free (SPF) environment at Suzhou Institute of Systems Medicine (ISM) under a controlled temperature (25°C) and a 12 h day‐night cycle. All animal experiments were conducted according to the US National Institutes of Health Guide for the Care and Use of Laboratory Animals and approved by the Animal Service Center of ISM (AUP no. ISM‐IACUC‐0011‐R).

### Reagents and antibodies

Anti‐α‐tubulin antibody (#T5168) were purchased from Sigma‐Aldrich (St. Louis, MO). PolyI:C, biotin‐polyI:C, polydA:dT, NF‐κB inhibitor BAY11‐7082, and MAPK inhibitors including SB203580, PD98059, and SP600125 were from InvivoGen (San Diego, CA). Recombinant mouse IFN‐α and IFN‐β were from PBL Assay Science (Piscataway, NJ). Recombinant human IFN‐β was from R&D system (Minneapolis, MN). Primary antibodies against GAPDH (#5174), β‐Actin (#3700), pSTAT1 (Tyr701, #9167), STAT1 (#14994), pTBK1 (Ser172, #5483), TBK1 (#3504), pIRF3 (Ser396, #29047), IRF3 (#4302), peIF2α (Ser51, #3398), eIF2α (#5324), Flag (#14793), and HRP‐linked secondary antibodies anti‐rabbit IgG (#7074) and anti‐mouse IgG (#7076) were from Cell Signaling Technology (Danvers, MA). Anti‐PKR (18244‐1‐AP) was purchased from Proteintech (Wuhan, China), anti‐IFIT2 (sc‐398610) was from Santa Cruz (Santa Cruz, CA). The fluorescence secondary antibodies were purchased from LI‐COR (Lincoln, NE).

### Primary cells and cell lines

BMDMs were harvested and differentiated from bone marrow cells of mice in DMEM supplemented with 10% heat‐inactivated fetal bovine serum (FBS), 1% penicillin/streptomycin (P/S), and 1% M‐CSF conditioned medium for 7 days. WT and *lincRNA‐EPS* ^−/−^ iBMMs were infected and immortalized by the J2 virus produced from cell line GG2EE (Palleroni *et al*, 1991; Ma *et al*, 2014), and cultured in RPMI1640 (10 mM HEPES pH 7.8, 10% heat‐inactivated FBS, 1% P/S, 1% M‐CSF conditioned medium). Conditioned medium containing M‐CSF was collected from the supernatant of GMG14‐12 cells (Gifted by Genhong Cheng Laboratory, University of California, Los Angeles). HEK293T, A549, and RAW264.7 were obtained from American Type Culture Collection (ATCC, Manassas, VA) and cultured in DMEM supplemented with 10% FBS and 1% P/S. For all experiments, cells were plated overnight, and the medium was replaced before stimulation, infection, or transfection. LincRNA‐EPS‐overexpressed and lincRNA‐EPS‐rescued iBMM cell lines were constructed using retroviruses packaged in HEK293T cells that transfected with pEco and pMSCV‐PIG‐lincRNA‐EPS (Gifted by Katherine A. Fitzgerald Laboratory, University of Massachusetts Medical School, Worcester). *LincRNA‐EPS*‐KD RAW264.7 cells were generated by using CRISPR/Cas9 technology. Briefly, a pair of sgRNAs (5′‐GAGCTCCAGGATGTCAGAAGAGG‐3′ and 5′‐ATGGTAAAACTACGTGTCAAAGG‐3′) targeting *lincRNA‐EPS* were cloned into the lentiCRISPRv2 vectors (Addgene, #52961). The constructed vectors were co‐transfected into WT RAW264.7 cell lines via electroporation using program D‐032 of Nucleofector 2b device (Lonza), following selected with 5 μg/ml puromycin at 24 h post‐transfection. The selected cells were cultured in the medium with 0.5 μg/ml puromycin and the expression of *lincRNA‐EPS* was confirmed by RT–qPCR.

### Cell transfection and stimulation

pMSCV‐PIG‐lincRNA‐EPS, pcDNA3.1‐PKR, and the corresponding empty vectors were transfected into HEK293T by using polyethylenimine (Merck). Plasmids were transfected into A549 cells and MEF by using Lipofectamine 2000 (Life Technologies) and JetPRIME (Polyplus Transfection) according to the manufacturer’s instructions, respectively. siRNAs targeting PKR and negative control were delivered into iBMMs with INTERFERin (Polyplus Transfection) according to the manufacturer’s instructions. PolyI:C and polydA:dT were transfected into RAW264.7, BMDMs, or iBMMs by Lipofectamine 2000. The ratio of transfection reagent to ligands was 2.5 (μl/μg). Cells were stimulated with LPS, IFN‐α, and IFN‐β as indicated in the figure legends.

### Viral infection and titer assay

The viruses VSV, VSV‐GFP, SeV, HSV‐1 (strain KOS), and Influenza (A/WSN) were gifted from Genhong Cheng Laboratory (University of California, Los Angeles). Before VSV, VSV‐GFP, SeV, and HSV‐1 infection, cultured cells in the regular medium were changed with medium containing 2% FBS, and then infected with different MOI as indicated in the figure legends. WSN was infected using specific medium containing 1 mg/ml TPCK‐E, 0.075% BSA, 1% P/S. 1 h post infection, the medium was replaced with regular medium containing 10% FBS and cultured for indicated times. For *in vivo* studies, littermate mice that age and sex‐matched were infected with VSV (6 × 10^7^ pfu/g, *i.v.*, 12 h) for detection of IFN‐β, virus titer in serum, and organs injuries. For survival study, littermate mice that age and sex‐matched were infected with VSV (1 × 10^8^ pfu/g, *i.v.*). The titer of VSV was measured using the methods of plaque assay for cell culture supernatant and TCID_50_ for serum (Ma *et al*, 2014; Lei *et al*, 2021).

### RNA extraction and real‐time quantitative PCR

Total RNA was extracted from cells using TRIzol (Thermo Fisher Scientific) according to the manufacturer’s instructions. After assessing RNA quality and concentration using NanoDrop 2000, equal amounts of RNA were reversed transcribed using PrimeScript RT Reagent Kit (Takara). RT–qPCR was performed using SYBR RT‐PCR kits (Takara). Gene expression levels were normalized to *Rpl32* as internal control genes by 2^−ΔΔCt^ cycle threshold method (Schmittgen & Livak, 2008). Primer sequences for RT–qPCR and ChIP‐qPCR are listed in Table EV2.

### RNA sequencing and data analysis

Total RNA for RNA sequencing was extracted from WT and *lincRNA‐EPS* ^−/−^ iBMMs in biological duplicates. After analyzed the quantity and quality of samples, RNA libraries were constructed using a TruSeq Stranded mRNA Sample Prep Kit (Illumina, San Diego, CA) according to the manufacturer’s guidelines. Then the libraries were sequenced on the HiSeq X10 using the paired‐end 2 × 150 bp, dual‐index format. For data analysis, CLC Genomics Workbench (Qiagen) was used to remove Illumina sequencing adapters within raw reads of every sample, and the clean reads were mapped to mouse mm 10 reference genome. Finally, differential genes (*P* ≤ 0.01, Fold change ≥ 2 or ≤ −2) were screened by CLC based on raw read counts. Heatmap analysis for RPKM of sequencing data was performed by R language using packages of pheatmap. RPKM of VSV‐infected WT and *lincRNA‐EPS* ^−/−^ iBMMs were analyzed by GSEA software to measure enrichment score for Hallmark data sets (Mootha *et al*, 2003; Subramanian *et al*, 2005). RNA sequencing raw data have been deposited in Gene Expression Omnibus (Accession no. GSE193326).

### Absolute copies quantification of lincRNA‐EPS

The RNA molecules of lincRNA‐EPS were produced by *in vitro* transcription according to the instructions of *in vitro* Transcription T7 Kit (Takara). LincRNA‐EPS was quantified by Nanodrop 2000 and subsequently diluted to perform RT–qPCR. The standard curve was illustrated based on RNA quantity, transcript length, and Ct value. Total RNA from a certain number of cells was isolated using TRIzol (Thermo Fisher Scientific) to determine the copy numbers of lincRNA‐EPS according to the standard curve.

### Western blot and RNA immunoprecipitation (RIP)

Cell lysates were prepared in lysis buffer containing 50 mM Tris–HCl (pH 7.5), 150 mM NaCl, 1% (v/v) NP‐40, and 0.5 mM EDTA supplemented with 1× complete protease inhibitor cocktail (Roche) and phosphatase inhibitor PhosSTOP (Roche). Equal total protein amounts from clarified lysates were resolved on SDS‐PAGE, and transferred to 0.2 μm PVDF membranes (Millipore) for Western blot analysis. The target proteins were visualized using ECL chemiluminescent substrate (Millipore) or Odyssey Imaging Systems (LI‐COR Biosciences). For RIP experiments, cell lysates were harvested in lysis buffer with 10 U RNase Inhibitor (Thermo Fisher Scientific), then performed as the introduction of Magna RIP Kit (Millipore) by 5 μg of anti‐PKR or 2 μg of anti‐flag antibodies with equal amount of isotype anti‐IgG, and using 20 μl Protein A/G Magnetic beads. Protein‐RNA complexes were treated with Proteinase K at 55°C for 30 min to elute and extract binding RNA. RNA samples were reverse transcribed and analyzed by qPCR. Results were normalized to input RNA extracted from cell lysates for RIP groups and isotype anti‐IgG groups.

### RNA pulldown assay and mass spectrometry

For RNA pulldown assay, RNA molecules were transcribed *in vitro* from amplified DNA fragments of lincRNA‐EPS flanked with T7 promoter sequence (5′‐TAATACGACTCACTATAGGG‐3′) at the 5′‐terminal according to the manufacturer’s instructions of *in vitro* Transcription T7 Kit (Takara). Transcribed RNA molecules of about 2,500 nt were confirmed by agarose gel electrophoresis and then ligated with a desthiobiotin linker using Pierce™ RNA 3′ End Desthiobiotinylation Kit (Thermo Fisher Scientific). The non‐labeled RNA in this kit was used as a negative control to ligate with the same linker. 50 pmol biotin‐lincRNA‐EPS, 1 μg biotin‐polyI:C, and matched amount of negative control molecules were performed to pulldown interacted proteins extracted from iBMM cell lysates with Pierce™ Magnetic RNA‐Protein Pull‐Down Kit (Thermo Fisher Scientific). Binding proteins that pulled down were loaded on SDS‐PAGE for silver staining (Thermo Fisher Scientific). The differential protein bands relative to negative control were sliced for mass spectrometry analysis by PTM BIO (China, Hangzhou).

### Chromatin immunoprecipitation (ChIP)

iBMM cells plated on 10‐cm dish were cross‐linked with 1% formaldehyde for 10 min at room temperature and quenched with 125 mM Glycine for 5 min. Nuclear pellets were isolated in hypotonic lysis buffer as indicated in the study of Atianand *et al* (2016), and vortex for 10 s every 5 min during 15 min incubation on ice. The nuclear pellets were suspended in the sonication buffer to incubate for 10 min on ice and then sonicated using Covaris S220 Focused‐ultrasonicator (peak incident power of 140 W, duty factor of 10%, treatment time of 60 s) to generate 200–500 bp chromatin fragments which were confirmed by agarose gel electrophoresis. Equal quantities of sheared chromatin (10 μg per IP) were used for IP assay with 2 µg anti‐H3K4me3 or isotype control IgG antibodies at 4°C overnight. The 20 μl Protein G Dynabeads (Invitrogen) were added in chromatin complexes at 4°C for 1 h and washed as indicated in the study of Atianand *et al* (2016). Purified DNA was analyzed by qPCR using primers designed at the transcriptional regulatory region of genes as described previously (Shen *et al*, 2012).

### Nuclear and cytoplasmic RNA fraction

A 5 × 10^7^ iBMM cell pellets were collected after washing with ice‐cold PBS twice and resuspended and lysed in 0.5 ml ice‐cold lysis buffer containing 0.15% NP‐40 and fractioned using 2.5 volumes of a chilled sucrose cushion (24% sucrose in lysis buffer) (Pandya‐Jones & Black, 2009). The nuclear and cytoplasmic fraction were dissolved in TRIzol‐LS (ThermoFisher Scientific) for RNA extraction and RT–qPCR.

### Statistical analysis

The data represent the mean of at least three independent experiments, and error bars represent the s.d. of the mean. Statistical analysis was performed by unpaired two‐tailed Student’s *t*‐test using GraphPad Prism (version 7; GraphPad Software Inc.). Survival data were analyzed using Log‐rank (Mantel‐Cox) test. *P* < 0.05 was considered as a statistically significant difference.

## Author contributions

**Jingfei Zhu:** Data curation; Investigation; Writing – original draft. **Shengchuan Chen:** Investigation. **Li‐Qiong Sun:** Investigation. **Siying Liu:** Data curation; Software; Investigation. **Xue Bai:** Investigation. **Dapei Li:** Investigation. **Fan Zhang:** Investigation. **Zigang Qiao:** Investigation. **Liang Li:** Investigation. **Haiping Yao:** Investigation. **Yu Xia:** Resources. **Ping Xu:** Resources; Funding acquisition. **Xiaohui Jiang:** Investigation. **Zhengrong Chen:** Resources. **Yongdong Yan:** Conceptualization; Supervision. **Feng Ma:** Conceptualization; Supervision; Funding acquisition; Writing – original draft; Writing – review & editing.

In addition to the CRediT author contributions listed above, the contributions in detail are:

FM and YY conceived the idea and designed the experiments. JZ, SC, LQS, SL, XB, DL, FZ, ZQ, LL, HY, and XJ performed all the experiments. YX, PX, and ZC provided the reagents and suggestions. FM and JZ analyzed the data and wrote the manuscript.

## Disclosure and competing interests statement

The authors declare that they have no conflict of interest.

## Supporting information



Expanded View Figures PDF

Table EV1

Table EV2

## Data Availability

The primary dataset of RNA‐seq have been deposited in Gene Expression Omnibus under the accession number GSE193326 (http://www.ncbi.nlm.nih.gov/geo/query/acc.cgi?acc=GSE193326). Sequence information for gene cloning and RNAi targets are available upon request.
