# Supplementary material for: LincRNA‐EPS impairs host antiviral immunity by antagonizing viral RNA–PKR interaction
Source: EMBO Rep. 2022 Mar 21;23(5):e53937. doi: 10.15252/embr.202153937 (PMC9066075; doi:10.15252/embr.202153937)
Supplement: Supplementary file 1 — Expanded View Figures PDF [file EMBR-23-e53937-s004.pdf]

Expanded View Figures

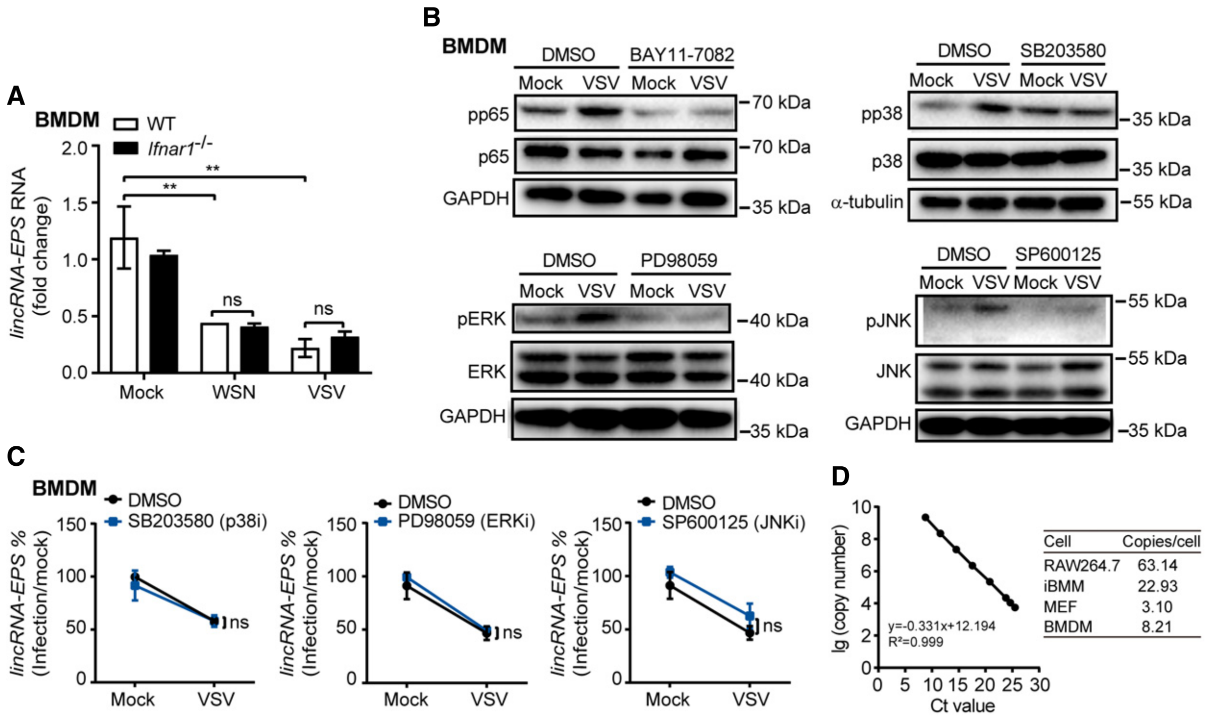

Figure EV1. The signaling pathways modulating lincRNA-EPS expression.

A RT–qPCR analysis of lincRNA-EPS transcripts in the WT and *Ifnar1*$^{-/-}$ BMDMs infected with WSN (MOI 1) and VSV (MOI 1) for 6 h.

B BMDMs were pretreated with NF-κB inhibitor BAY11-7082 (1 μM), p38 MAPK inhibitor SB203580 (5 μM), ERK inhibitor PD98059 (50 μM) and JNK inhibitor SP600125 (20 μM) for 1 h prior and infected with VSV (MOI 1) for 6 h. The phosphorylation and total proteins were detected by Western blot. GAPDH and α-tubulin were shown as loading control.

C The percentage of lincRNA-EPS transcripts downregulated in the VSV-infected group compared with Mock group were calculated.

D Copy-number analysis of lincRNA-EPS transcripts in several cell types by RT–qPCR. Standard curve was generated using *in vitro* transcribed RNA molecule of lincRNA-EPS as template.

Data information: Data of (A, C) are shown as the mean ± s.d. from three independent experiments, $^{**}P < 0.01$ and ns, not significant by unpaired Student's *t*-test. Data of (B, D) are representative results from three independent experiments.

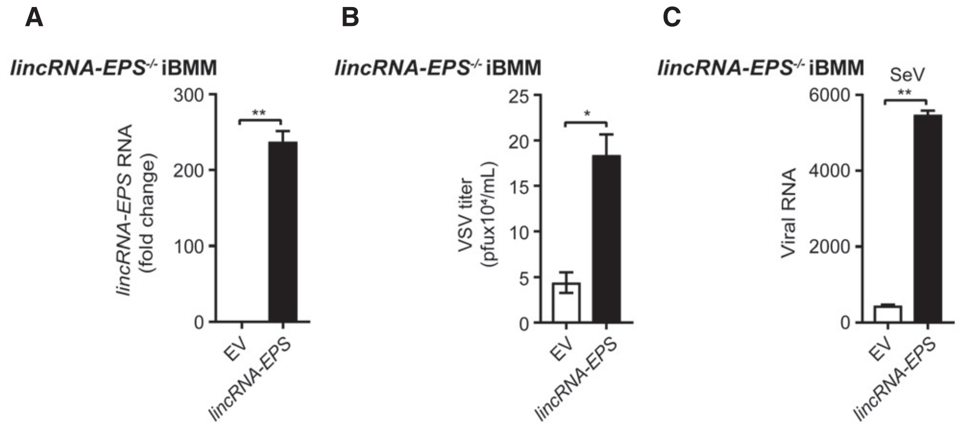

Figure EV2.

 © 2022 The Authors

◀ **Figure EV2. Rescued expression of lincRNA-EPS inhibits host antiviral ability.**

A The lincRNA-EPS transcripts were measured by RT–qPCR in the control (EV) and lincRNA-EPS-rescued (*lincRNA-EPS*) iBMMs.

B, C LincRNA-EPS-rescued iBMMs and control group were infected with VSV (MOI 1) and SeV (MOI 1) for 12 h, VSV titers were measured by plaque assay (B) and SeV RNA level were checked by RT–qPCR (C).

Data information: All data are shown as the mean ± s.d. from three independent experiments, *$P < 0.05$ and **$P < 0.01$ by unpaired Student's *t*-test.

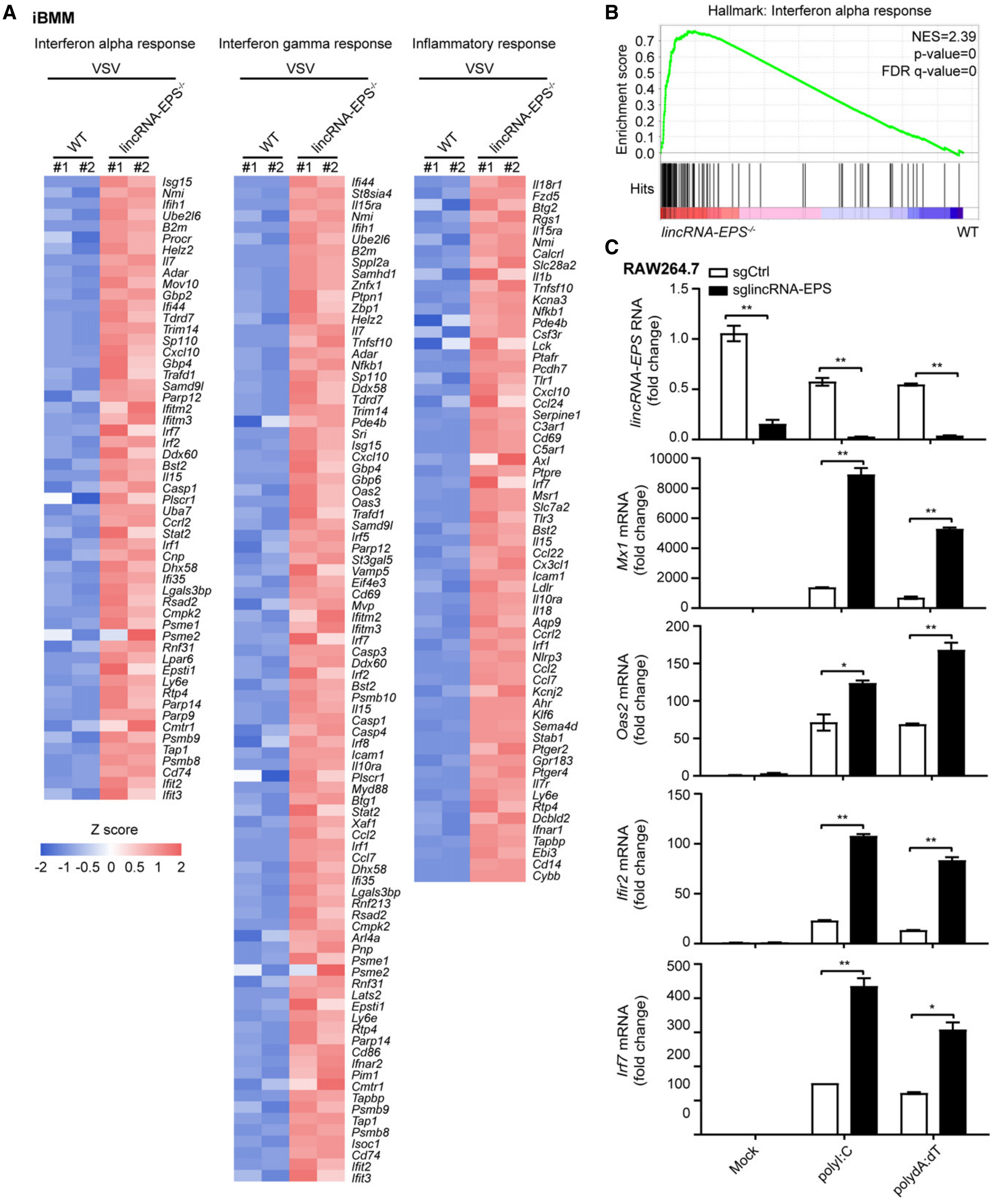

Figure EV3.

© 2022 The Authors

Figure EV3. Greater induction of antiviral ISGs in the *lincRNA-EPS*$^{-/-}$ macrophages.

A Heatmap analysis of differential genes expression in top three pathways of GSEA.
B Enrichment plot of Interferon alpha response was extracted from GSEA.
C Control (sgCtrl) and lincRNA-EPS knockdown (sglincRNA-EPS) RAW264.7 cells were transfected with 1 μg/ml polyI:C and polydA:dT for 10 h, the transcripts of *lincRNA-EPS* and *Mx1*, *Oas2*, *Ifit2*, and *Irf7* were measured by RT–qPCR.

Data information: Data of (C) are shown as the mean ± s.d. from three independent experiments, $*P < 0.05$ and $**P < 0.01$ by unpaired Student's *t*-test.

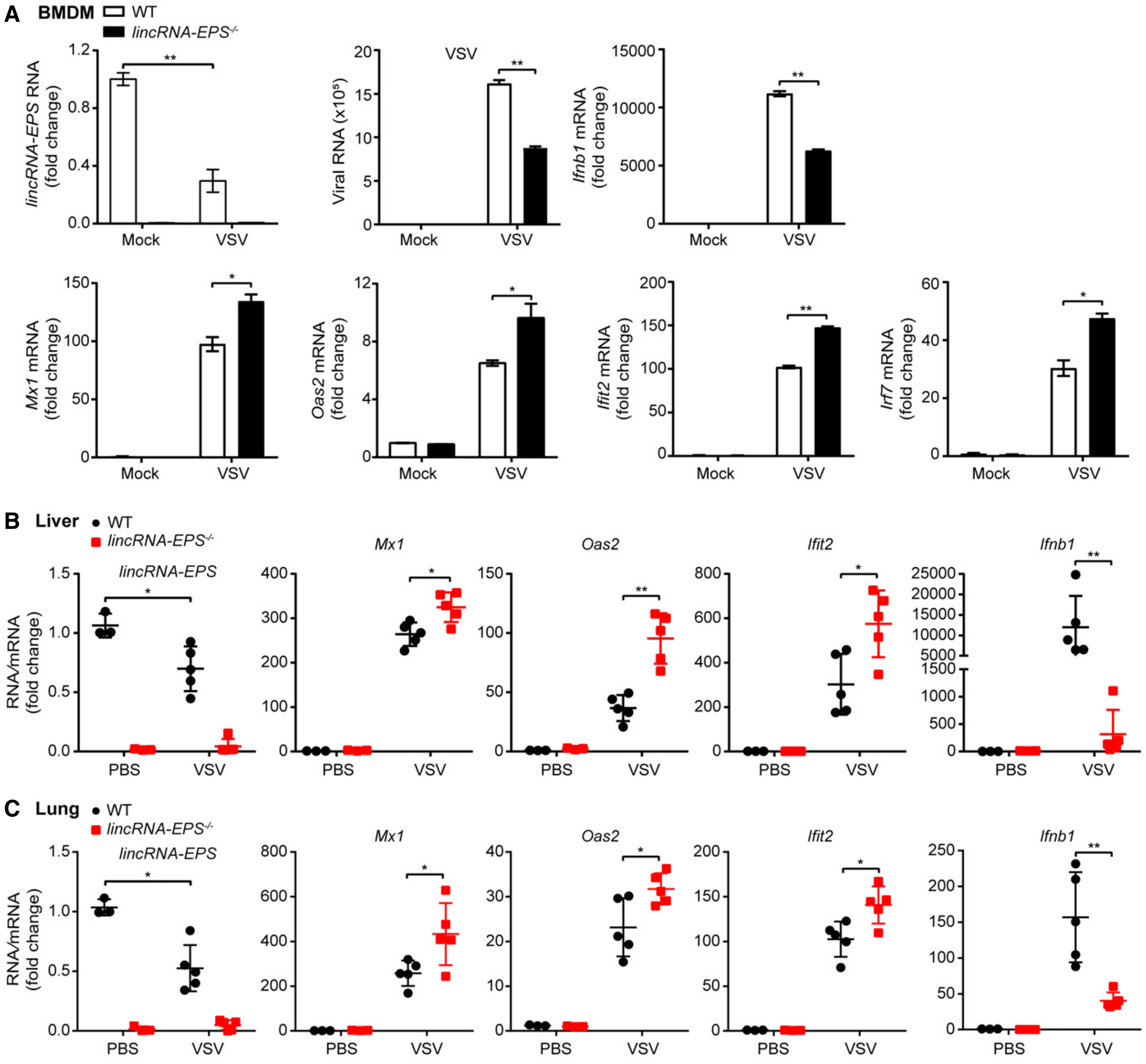

Figure EV4.

◀ **Figure EV4. Deficiency of lincRNA-EPS enhanced ISGs expression in primary macrophages and infected mice model.**

A RT–qPCR analysis of *lincRNA-EPS* transcripts, viral RNA and *Ifnb1, Mx1, Oas2, Ifit2, Irf7* mRNA level in the WT and *lincRNA-EPS*$^{-/-}$ BMDMs infected with VSV (MOI 5) for 10 h.

B, C Eight weeks female *lincRNA-EPS*$^{-/-}$ mice ($n = 5$) and WT littermates ($n = 5$) were injected (*i.v.*) with VSV (sub-lethal dose, 6×10^7 pfu/g) for 12 h, and negative control groups were injected with PBS ($n = 3$). RT–qPCR analysis of *lincRNA-EPS* transcripts and *Mx1, Oas2, Ifit2, Ifnb1* mRNA level from liver (B) and lung (C) tissue homogenate.

Data information: Data of (A) are shown as the mean ± s.d. from three independent experiments, data of (B, C) are shown as the mean ± s.d. of a typical representative result from three independent experiments, and one dot represents a mouse. *$P < 0.05$ and **$P < 0.01$ by unpaired Student's *t*-test.

 © 2022 The Authors

22 of 17 EMBO reports 23: e53937 | 2022 © 2022 The Authors

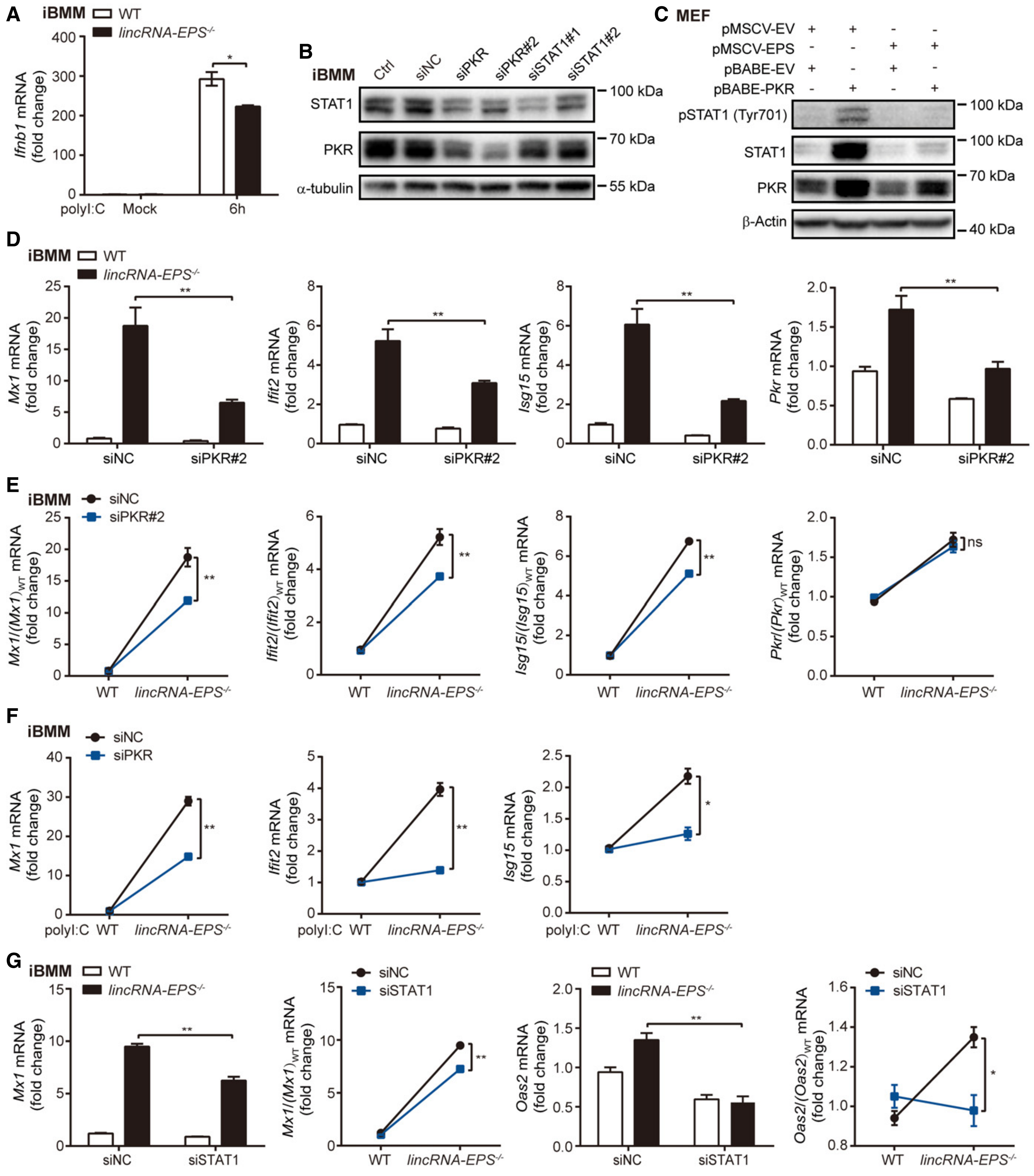
A
iBMM
WT
lincRNA-EPS-/-
Ifnb1 mRNA (fold change)
polyI:C Mock 6h
B
iBMM
Ctrl siNC siPKR siPKR#2 siSTAT1#1 siSTAT1#2
STAT1
PKR
α-tubulin
100 kDa
70 kDa
55 kDa
C MEF
pMSCV-EV
pMSCV-EPS
pBABE-EV
pBABE-PKR
pSTAT1 (Tyr701)
STAT1
PKR
β-Actin
100 kDa
70 kDa
40 kDa
D
iBMM
Mx1 mRNA (fold change)
Ifit2 mRNA (fold change)
Isg15 mRNA (fold change)
Pkr mRNA (fold change)
siNC siPKR#2
E
siNC
siPKR#2
Mx1/(Mx1)WT mRNA (fold change)
Ifit2/(Ifit2)WT mRNA (fold change)
Isg15/(Isg15)WT mRNA (fold change)
Pkr/(Pkr)WT mRNA (fold change)
ns
WT lincRNA-EPS-/-
F
siNC
siPKR
polyI:C WT lincRNA-EPS-/-
G
siSTAT1
Oas2 mRNA (fold change)
Oas2/(Oas2)WT mRNA (fold change)

Figure EV5.

Figure EV5. LincRNA-EPS inhibits PKR-STAT1-dependent induction of ISGs.

A RT–qPCR analysis of *Ifnb1* mRNA level of polyI:C (1 μg/ml) transfected iBMMs for 6 h.

B 20 nM siRNA for negative control (NC), PKR and STAT1 were transfected into iBMMs for 48 h. STAT1 and PKR protein level relative to naïve cells (Ctrl) were detected by Western blot and α-tubulin was shown as a loading control.

C Empty vector (pMSCV-EV) and pMSCV-PIG-lincRNA-EPS (pMSCV-EPS) were transfected into PKR-overexpressed MEF cell lines (pBABE-PKR) and control cells (pBABE-EV), phosphorylation and total levels of STAT1 and PKR were detected by Western blot and β-Actin was shown as a loading control.

D, E 20 nM siRNA for NC (siNC) and PKR (siPKR#2) were transfected into WT and *lincRNA-EPS*$^{-/-}$ iBMMs, *Mx1, Ifit2, Isg15 and Pkr* mRNA level were measured by RT–qPCR (D), and relative basal expression change rates of the antiviral ISGs in *lincRNA-EPS*$^{-/-}$ iBMMs were calculated compared with WT group (E).

F 20 nM siRNA for NC and PKR were transfected into WT and *lincRNA-EPS*$^{-/-}$ iBMMs following transfected with 1 μg/ml polyI:C for 6 h, *Mx1, Ifit2, Isg15* mRNA level were measured by RT–qPCR. Relative expression change rates of ISGs in *lincRNA-EPS*$^{-/-}$ iBMMs were calculated compared with WT group.

G 20 nM siRNA for NC and STAT1 were transfected into WT and *lincRNA-EPS*$^{-/-}$ iBMMs, *Mx1, Oas2* mRNA level were measured by RT–qPCR. Relative expression change rates of ISGs in *lincRNA-EPS*$^{-/-}$ iBMMs were calculated compared with WT group.

Data information: Data of (A, and D–G) are shown as the mean ± s.d. from three independent experiments, *$P < 0.05$ and **$P < 0.01$ by unpaired Student's *t*-test. Data of (B–C) are representative images from three independent experiments.

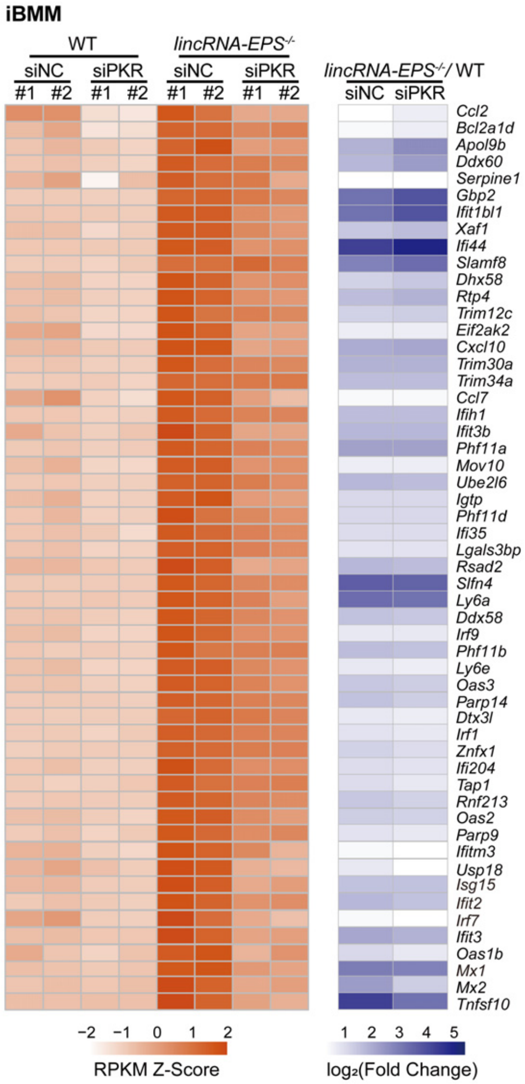

Figure EV6. Downregulated ISGs after interfering PKR expression.

20 nM siRNAs for negative control (siNC) and PKR (siPKR) were transfected into iBMMs for 48 h, and the total RNA was isolated to perform RNA sequencing. The mean RPKM values of the biological duplicates were calculated and the downregulated antiviral ISGs after knocking down PKR were listed by heatmap (left panel). The fold change of ISGs level of lincRNA-EPS$^{-/-}$ iBMMs comparing to the WT iBMMs in siNC and siPKR groups were also illustrated by heatmap (right panel).

 © 2022 The Authors
