# Supplementary material for: LincRNA‐EPS impairs host antiviral immunity by antagonizing viral RNA–PKR interaction
Source: EMBO Rep. 2022 Mar 21;23(5):e53937. doi: 10.15252/embr.202153937 (PMC9066075; doi:10.15252/embr.202153937)
Supplement: Supplementary file 4 [file EMBR-23-e53937-s003.pdf]

EMBO reports

# LincRNA-EPS impairs host antiviral immunity by antagonizing viral RNA-PKR interaction

Jingfei Zhu, Shengchuan Chen, Li-Qiong Sun, Siying Liu, Xue Bai, Dapei Li, Fan Zhang, Zigang Qiao, Liang Li, Haiping Yao, Yu Xia, Ping Xu, Xiaohui Jiang, Zhengrong Chen, Yongdong Yan, and Feng Ma
**DOI: 10.15252/embr.202153937**

*Corresponding author(s): Feng Ma (maf@ism.pumc.edu.cn) , Yongdong Yan (yyd3060@126.com)*

---

## Review Timeline:

| | |
|---|---|
| Submission Date: | 3rd Sep 21 |
| Editorial Decision: | 8th Oct 21 |
| Revision Received: | 29th Jan 22 |
| Editorial Decision: | 1st Mar 22 |
| Revision Received: | 3rd Mar 22 |
| Accepted: | 7th Mar 22 |

---

*Editor: Esther Schnapp*

## Transaction Report:

(Note: With the exception of the correction of typographical or spelling errors that could be a source of ambiguity, letters and reports are not edited. Depending on transfer agreements, referee reports obtained elsewhere may or may not be included in this compilation. Referee reports are anonymous unless the Referee chooses to sign their reports.)

**1st Editorial Decision** **8th Oct 2021**

Dear Dr. Ma,

Thank you for the submission of your manuscript to EMBO reports. We have now received the full set of referee reports as well as referee cross-comments that are pasted below.

As you will see, the referees acknowledge that the findings are potentially interesting. However, they also point out that significant revisions will be required before the study can be considered for publication here. The referees note that the link between lincRNA-EPs and PKR is weak, that the role of STAT1 should be examined, that it should be clarified which genes are PKR-STAT1 regulated and which genes are hnRNPL-EPS controlled, that primary BMDM from lincRNA-EPS deficient mice should be used, and that an endogenous interaction of lincRNA-EPS with PKR should be shown. We agree that all these are important points, thus all referee concerns should be addressed. If you like, we can also talk about the revision requirements in a video chat.

If you decide to embark on such a revision, I would like to invite you to revise your manuscript with the understanding that the referee concerns must be fully addressed and their suggestions taken on board. Please address all referee concerns in a complete point-by-point response. Acceptance of the manuscript will depend on a positive outcome of a second round of review. It is EMBO reports policy to allow a single round of major revision only and acceptance or rejection of the manuscript will therefore depend on the completeness of your responses included in the next, final version of the manuscript.

Revised manuscripts should be submitted within three months of a request for revision; they will otherwise be treated as new submissions. Please contact us if a 3-months time frame is not sufficient for the revisions so that we can discuss this further. You can either publish the study as a short report or as a full article. For short reports, the revised manuscript should not exceed 27,000 characters (including spaces but excluding materials & methods and references) and 5 main plus 5 expanded view figures. The results and discussion sections must further be combined, which will help to shorten the manuscript text by eliminating some redundancy that is inevitable when discussing the same experiments twice. For a normal article there are no length limitations, but it should have more than 5 main figures and the results and discussion sections must be separate. In both cases, the entire materials and methods must be included in the main manuscript file.

IMPORTANT NOTE: we perform an initial quality control of all revised manuscripts before re-review. Your manuscript will FAIL this control and the handling will be DELAYED if the following APPLIES:
1) A data availability section providing access to data deposited in public databases is missing. If you have not deposited any data, please add a sentence to the data availability section that explains that.
2) Your manuscript contains statistics and error bars based on n=2. Please use scatter blots in these cases. No statistics should be calculated if n=2.

When submitting your revised manuscript, please carefully review the instructions that follow below. Failure to include requested items will delay the evaluation of your revision.

1) a .docx formatted version of the manuscript text (including legends for main figures, EV figures and tables). Please make sure that the changes are highlighted to be clearly visible.

2) individual production quality figure files as .eps, .tif, .jpg (one file per figure). See https://wol-prod-cdn.literatumonline.com/pb-assets/embo-site/EMBOPress_Figure_Guidelines_061115-1561436025777.pdf for more info on how to prepare your figures.

3) We replaced Supplementary Information with Expanded View (EV) Figures and Tables that are collapsible/expandable online. A maximum of 5 EV Figures can be typeset. EV Figures should be cited as 'Figure EV1, Figure EV2" etc... in the text and their respective legends should be included in the main text after the legends of regular figures.

- For the figures that you do NOT wish to display as Expanded View figures, they should be bundled together with their legends in a single PDF file called *Appendix*, which should start with a short Table of Content. Appendix figures should be referred to in the main text as: "Appendix Figure S1, Appendix Figure S2" etc. See detailed instructions regarding expanded view here: <https://www.embopress.org/page/journal/14693178/authorguide#expandedview>

- Additional Tables/Datasets should be labeled and referred to as Table EV1, Dataset EV1, etc. Legends have to be provided in a separate tab in case of .xls files. Alternatively, the legend can be supplied as a separate text file (README) and zipped together with the Table/Dataset file.

4) a .docx formatted letter INCLUDING the reviewers' reports and your detailed point-by-point responses to their comments. As part of the EMBO Press transparent editorial process, the point-by-point response is part of the Review Process File (RPF), which will be published alongside your paper.

5) a complete author checklist, which you can download from our author guidelines

<https://www.embopress.org/page/journal/14693178/authorguide>. Please insert information in the checklist that is also reflected in the manuscript. The completed author checklist will also be part of the RPF.

6) Please note that all corresponding authors are required to supply an ORCID ID for their name upon submission of a revised manuscript (<https://orcid.org/>). Please find instructions on how to link your ORCID ID to your account in our manuscript tracking system in our Author guidelines
<https://www.embopress.org/page/journal/14693178/authorguide#authorshipguidelines>

7) Before submitting your revision, primary datasets produced in this study need to be deposited in an appropriate public database (see https://www.embopress.org/page/journal/14693178/authorguide#datadeposition). Please remember to provide a reviewer password if the datasets are not yet public. The accession numbers and database should be listed in a formal "Data Availability" section placed after Materials & Method (see also
https://www.embopress.org/page/journal/14693178/authorguide#datadeposition). Please note that the Data Availability Section is restricted to new primary data that are part of this study. * Note - All links should resolve to a page where the data can be accessed. *
If your study has not produced novel datasets, please mention this fact in the Data Availability Section.

8) We would also encourage you to include the source data for figure panels that show essential data. Numerical data should be provided as individual .xls or .csv files (including a tab describing the data). For blots or microscopy, uncropped images should be submitted (using a zip archive if multiple images need to be supplied for one panel). Additional information on source data and instruction on how to label the files are available at
<https://www.embopress.org/page/journal/14693178/authorguide#sourcedata>.

9) Our journal also encourages inclusion of *data citations in the reference list* to directly cite datasets that were re-used and obtained from public databases. Data citations in the article text are distinct from normal bibliographical citations and should directly link to the database records from which the data can be accessed. In the main text, data citations are formatted as follows: "Data ref: Smith et al, 2001" or "Data ref: NCBI Sequence Read Archive PRJNA342805, 2017". In the Reference list, data citations must be labeled with "[DATASET]". A data reference must provide the database name, accession number/identifiers and a resolvable link to the landing page from which the data can be accessed at the end of the reference. Further instructions are available at https://www.embopress.org/page/journal/14693178/authorguide#referencesformat

10) Regarding data quantification (see Figure Legends:
https://www.embopress.org/page/journal/14693178/authorguide#figureformat)

The following points must be specified in each figure legend:

- the name of the statistical test used to generate error bars and P values,

- the number (n) of independent experiments (please specify technical or biological replicates) underlying each data point,

- the nature of the bars and error bars (s.d., s.e.m.),

- If the data are obtained from n {less than or equal to} 2, use scatter blots showing the individual data points.

Discussion of statistical methodology can be reported in the materials and methods section, but figure legends should contain a basic description of n, P and the test applied.

- Please also include scale bars in all microscopy images.

We would also welcome the submission of cover suggestions, or motifs to be used by our Graphics Illustrator in designing a cover.

As part of the EMBO publication's Transparent Editorial Process, EMBO reports publishes online a Review Process File (RPF) to accompany accepted manuscripts. This File will be published in conjunction with your paper and will include the referee reports, your point-by-point response and all pertinent correspondence relating to the manuscript.

You are able to opt out of this by letting the editorial office know (emboreports@embo.org). If you do opt out, the Review Process File link will point to the following statement: "No Review Process File is available with this article, as the authors have chosen not to make the review process public in this case."

I look forward to seeing a revised form of your manuscript when it is ready. Please use this link to submit your revision:
https://embor.msubmit.net/cgi-bin/main.plex

Yours sincerely,

Esther Schnapp, PhD
Senior Editor
EMBO reports

Referee #1:

In this manuscript, Zhu et al. investigate the expression of lincRNA-EPS following viral infection of mouse BMDMs and discovered that it was rapidly down-regulated (Fig. 1). Type-I IFN treatment of the cells also promoted this decrease, but the decrease was still seen in Ifnar1 KO cells (albeit modestly decreased) indicating that the effect was partially through another pathway - which the authors propose to be NFkB using a broad inhibitor. The authors go on to demonstrate that overexpression (12 fold) favors viral infecion while CRISPR CAS-9 in RAW cells decreased infection. The authors confirmed these observations in immortalised BMDMS from EPS-KO mice, which were protected against VSV in vivo. Importantly, the mice lacking EPS exhibited less VSV and decreased IFNb levels. RNAseq signature revealed that several ISGs were increased upon EPS deficiency basally and upon infection (and decreased upon overexpression). Deficiency in EPS lead to basal increased transcriptional activity of MX1 and OAS2, and was associated with increased STAT1 phosphorylation. The authors then show evidence that PKR binds EPS and that RNAi of PKR alleviates some of the antiviral effects of EPS-/-. This leads the authors to conclude that EPS is a key regulator of antiviral responses through its modulation of PKR-driven STAT1.
Overall there are several key issues that need to be addressed to clarify the line of thought of the manuscript and support its conclusions and novelty.

Main points:
1- The observation that EPS is rapidly decreased upon infection with VSV is not very surprising given that this has previously been observed with pIC and SeV (Atianand et al 2016). That specific Atianand paper also showed that EPS deletion indeed lead to basal upregulation of several key ISGs (indeed highlighted in Fig 5E of this paper), with several having direct antiviral effects (such as RSAD2, MX1, IFIT1). To this reviewer it is hard to see what the first 5 figures of the current paper really add to this previous knowledge aside from the in vivo experiment (we already knew that EPS deficiency was boosting select ISG expression, so it seems incremental to see that there is less viral replication with the viruses shown). There are several issues in claiming that IFNb is not regulated (since basally it is actually up in EPS KO) when using a viral infection. The data from figure 4E should be complemented by looking at IFNb induction with pIC and pdAdT; similarly the WB fig5H should be repeated with pIC as an inducer rather than the virus (since the viral replication is altered due to basal ISG expression).

2- The link with PKR is tenuous. This reviewer notes that there is an estimated 11 copies of EPS RNA per cell (based on the Cell paper cited above), and it is hard to imagine that these molecules would be naturally interacting with PKR when they are principally bound to nuclear chromatin (this needs to be discussed). Critically, most of the data linking PKR is based on RNAi with a single siRNA. Given the off-target effects of RNAi, it is essential to have at least two independent siRNAs targeted to the same target and showing the same phenotype to support any conclusions made. The theory of the authors does not explain how in the absence of infection, the ISG (STAT1 targets) they picked would be induced by the PKR-STAT1 axis (noting that STAT1-P is entirely absent in mock cells Fig 5H). Critically, if the PKR-STAT1 axis is really involved, then RNAi of STAT1 should also decrease the basal expression of selected ISGs basally engaged in EPS cells.

3- The authors entirely omit the concept presented in the
Atianand paper that EPS Maintains a Repressed Chromatin State at selected IRGs (including key antiviral genes) - which directly explains why some antiviral genes are basally up-regulated in EPS KO cells. Which genes would be PKR-STAT1 regulated and which genes would be hnRNPL-EPS controlled??? Since many antiviral genes such as RSAD2 and IFIT1 were found to be regulated by the hnRNPL-EPS axis it is hard to distinguish the antiviral effects of the proposed PKR-STAT1 axis (but this is an essential thing to perform experimentally to substantiate the overall point of the paper).

Minor:
- Fig 2A/B... what is the biological relevance of a 12 fold increase of EPS RNA???
- Fig 5F/G is missing color keys
- Why are you using immortalised BMDMs from EPS-/- mice (which end up being similar to your RAW cells) and not primary cells?
- Fig 6F should be relative to each siRNA (siPKR increased viral load in WT so cannot be 1).

Referee #2:

lincRNA-EPS has been previously characterized as an immunoregulatory lncRNA that restrains inflammatory responses,

although its role in antiviral immunity is unknown. Here the demonstrate that lincRNA-EPS as a negative regulator of host antiviral responses. They report that lincRNA-EPS is downregulated during RNA virus infection, to allow an adequate anti-viral response. The authors demonstrate that LincRNA-EPS binds PKR to block its interaction with viral RNA. Blockade of the PKR-viral RNA interaction impairs activation of STAT1 and ISG expression rendering mice more susceptible to RNA virus infection. The results are interesting and add to our knowledge of lncEPS and lncRNA dependent regulation of antiviral immunity more broadly.

There is an overreliance on immortalized cells and overexpression systems. The paper would be strengthened if some of the key observaions could be validated in primary cells and an endogenous interaction of lincRNA-EPS and PKR examined.

Major comments:
1. Figure 1J should include an analysis of absolute copy number of lincRNA-EPS. RNA FISH analysis could also be highly informative here.
2. Throughout the figures are 1 experiment. Instead they should include the mean of all independent experiments performed.
3. It's unclear why immortalized cells were used in Figure 3-5 rather than primary BMDM from lincRNA-EPS deficient mice and WT littermate controls. The main findings of these figures should be repeated using primary BMDMs.
4. The decrease in viral titer in the serum of lincRNA-EPS mice (Figure 3J) is very modest when compared to WT and is not consistent with the robust phenotype observed in Figure 3G. It would be beneficial to assess the viral titer in the liver and lungs after infection.
5. Figure 4 should include protein levels of at least some representative ISGs.
6. The authors should include a full list of lincRNA-EPS interacting proteins identified by mass spectrometry. Additional controls such as the use of biotinylated lincRNA-EPS probes in lincRNA-EPS deficient BMDMs would further strengthen this part of the study.
7. Figure 7C-G demonstrate upregulated expression of ISGs in mock cells deficient in lincRNA-EPS when compared to WT cells. However, no phospo-STAT1 was detected in lincRNA-EPS KI cells (Figure 5H). The authors should explain this discrepancy.
8. Figure 7I: a better explanation of the proposed mechanism including subcellular localization should be provided.

Minor comments:
1. Figure 4A: The authors should provide a heatmap demonstrating a full list of genes and pathways detected by the assay.
2. Figure 5F is missing a legend.
3. Figure 6D should include detection of PKR in naïve cells as an additional negative control.
4. Figure 7A: Can the author explain why total STAT1 is downregulated in PKR deficient cells?

Referee #3:

In this study, Zhu et al. describe a role of LincRNA-EPS in the negative regulation of STAT1-triggered type I IFN responses. PKR is well-known as a nucleic acids receptor of viral dsRNA. PKR activity needs to be restricted in the resting state to prevent autoimmune activation. The authors claim that lincRNA-EPS binds to PKR and antagonizes the viral RNA-PKR interaction and then inhibits PKR-induced STAT1 activation, resulted in the suppression of downstream ISGs production. Virus infection significantly decreases LincRNA-EPS expression and subsequently removes the restrictions of PKR activation. Therefore, this study help us to understand the self-limiting of the innate immune response. Overall, the findings are novel and interesting. The experiments are well performed, and the manuscript is fluent and generally well organized. However, some concerns mentioned below must be addressed before the manuscript can be considered for publication.

Major comments:
1.As the author describes that PKR is required for phosphorylation and activation of STAT1 under the response to IFN-γ and LPS, but PKR or LincRNA-EPS inhibition greatly changed the total protein level of STAT1(Fig.5H, Fig.6A and 6B). Activation and phosphorylation of STAT1 should not affect its total protein level, is there any other mechanism for regulation of STAT1?
2.In Fig. 7C, 7E and 7G, WT-siPKR almost blocked VSV infection-induced ISGs expression, but LincRNA-EPS-/--siPKR greatly enhanced ISGs expression compared with WT-siPKR. Therefore PKR knockdown could not change the positive effect of lincRNA-EPS knockout on ISG formation, these results strongly support the existence of other mechanisms that regulate JAK-STAT signaling by LincrNA-EPS. The authors should use IFN-β stimulation instead of VSV infection to investigate whether this phenomenon is caused by viral infection specificity.
3.The H3K4me3 is associated with high transcription activity. However, LincrNA-EPS knockout does not affect the IFN-β induced H3K4me3 of ISGs in the activation state but not the rest state(Fig. 5G and 5H). What are the reasons for the different effect ?
4.The authors claim that lincRNA-EPS regulates STAT1 activation and subsequently controls ISGs expression. However, the study only examined the effects of lincRNA-EPS on virus replication and tissue damage in vivo. Whether lincRNA-EPS could

control ISGs expression in tissues (such as lung, spleen or liver) or serum?
5.In Fig. 6H and 6I, lincRNA-EPS overexpression competitively binds virus RNA, which in turn inhibits PKR activation, but more evidence is needed to support this conclusion. Whether lincRNA-EPS knockout or knockdown could promote PKR binds RNA? Whether other dsRNA(such as poly(I: C) have similar phenomena to the VSV-RNA?

Minor comments:
1.The graphic symbols are missing in Fig.5F and 5G.
2.Some description of the background of VSV-N and G is required.
3.In Fig. 3G, the survival percentage of the lincRNA-EPS knockout group did not appear to be calculated from 100%.

Further comments from referee 1:

If the authors conducted an RNAseq experiment of EPS-WT and EPS-KO after PKR down-regulation, it may help provide insights into the claimed PKR/EPS-dependent ISG signature - which should only be a portion of the ISGs see in EPS-WT. KO or down-regulation of such PKR-dependent ISGs in the context of EPS-KO cells with infection would be the only direct way to link EPS-PKR targets and antiviral effects.

Dear Dr. Esther Schnapp,

We are submitting our revised manuscript entitled "LincRNA-EPS negatively regulates host antiviral immunity by antagonizing viral RNA interacting with PKR" (EMBOR-2021-53937V1) to ***EMBO Reports*** for consideration of publication.

We appreciate all the comments and suggestions from you and all three reviewers. According to the comments and suggestions, we have revised our manuscript by adding new experimental data (**Figures 3F, 3I, 3J, 4B, 4D, 5G, 5H, 5I, 6B, 6C, 6I, and Figures EV1D, EV3A, EV4, EV5)** and editing our manuscript carefully. The RNA-Seq data analysis (**Appendix Figure S1**) have been also included. We have provided point-by-point response as below and indicated the changes (yellow highlighted) in our revised manuscript.

We think that we have addressed all the concerns raised by you and the reviewers. As a result, the manuscript is indeed significantly improved. Thank you very much for your consideration. We are looking forward to hearing from you.

With my best regards

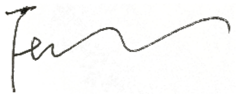

Feng Ma, M.D.

Institute of Systems Medicine, Chinese Academy of Medical Sciences

100 Chongwen Road, Suzhou Industry Park

Suzhou215123, Jiangsu, China

**Subject:** EMBOR-2021-53937V1 Decision Letter

*As you will see, the referees acknowledge that the findings are potentially interesting. However, they also point out that significant revisions will be required before the study can be considered for publication here. The referees note that the link between lincRNA-EPS and PKR is weak, that the role of STAT1 should be examined, that it should be clarified which genes are PKR-STAT1 regulated and which genes are hnRNPL-EPS controlled, that primary BMDM from lincRNA-EPS deficient mice should be used, and that an endogenous interaction of lincRNA-EPS with PKR should be shown. We agree that all these are important points, thus all referee concerns should be addressed. If you like, we can also talk about the revision requirements in a video chat.*

**Response**: Thank you for the opportunity to revise our manuscript. To address all the concerns raised by the reviewers, we have performed new experiments and added the results in the Figures 3F, 3I, 3J, 4B, 4D, 5G, 5H, 5I, 6B, 6C, 6I, EV1D, EV3A, EV4, and EV5 of the revised manuscript. Point-by-point response to each question has been provided. In addition, RNA-Seq analysis was also performed to distinguish the PKR-STAT1 regulated genes and the hnRNPL-EPS controlled genes (Appendix Figure S1).

==============================================================

Referee #1:

*In this manuscript, Zhu et al. investigate the expression of lincRNA-EPS following viral infection of mouse BMDMs and discovered that it was rapidly down-regulated (Fig 1). Type-I IFN treatment of the cells also promoted this decrease, but the decrease was still seen in Ifnar1 KO cells (albeit modestly decreased) indicating that the effect was partially through another pathway - which the authors propose to be NF-kB using a broad inhibitor. The authors go on to demonstrate that overexpression (12-fold) favors viral infection while CRISPR CAS-9 in RAW cells decreased infection. The authors confirmed these observations in immortalized BMDMS from EPS-KO mice, which were protected against VSV in vivo. Importantly, the mice lacking EPS exhibited less VSV and decreased IFNb levels. RNA-seq signature revealed that several ISGs were increased upon EPS deficiency basally and upon infection (and decreased upon overexpression). Deficiency in EPS lead to basal increased transcriptional activity of MX1 and OAS2, and was associated with increased STAT1 phosphorylation. The authors then show evidence that PKR binds EPS and that RNAi of PKR alleviates some of the antiviral effects of $EPS^{-/-}$. This leads the authors to conclude that EPS is a key regulator of antiviral responses through its modulation of PKR-driven STAT1. Overall, there are several key issues that need to be addressed to clarify the line of thought of the manuscript and support its conclusions and novelty.*

**Response**: We appreciate your evaluation and valuable comments for our manuscript. According to your suggestions, we have revised our manuscript by adding new experimental data (Figure 5I, EV1D, EV4, EV5, and Appendix Figure S1) and editing our manuscript carefully. Hopefully we have addressed all your concerns. Point-by-point response to your question has been provided as below.

Main points:

*1- The observation that EPS is rapidly decreased upon infection with VSV is not very surprising given that this has previously been observed with pIC and SeV (Atianand et al 2016). That specific Atianand paper also showed that EPS deletion indeed lead to basal upregulation of several key ISGs (indeed highlighted in Fig 5E of this paper), with several having direct antiviral effects (such as RSAD2, MX1, IFIT1). To this reviewer it is hard to see what the first 5 figures of the current paper really add to this previous knowledge aside from the in vivo experiment (we already knew that EPS deficiency was boosting select ISG expression, so it seems incremental to see that there is less viral replication with the viruses shown). There are several issues in claiming that IFNb is not regulated (since basally it is actually up in EPS KO) when using a viral infection. The data from figure 4E should be complemented by looking at IFNb induction with pIC and pdAdT; similarly, the WB fig5H should be repeated with pIC as an inducer rather than the virus (since the viral replication is altered due to basal ISG expression).*

**Response**: Thanks for your comments and suggestion. We admit that the study by Atianand *et al.* (PMID: 27315481) inspirated us and drove us to investigate the detail function of *lincRNA-EPS* in antiviral immunity. In addition to the repressive role of *lincRNA-EPS* in inflammation described by Atianand *et al*, our current study not only has demonstrated that *lincRNA-EPS* inhibits host antiviral immunity *in vitro* and *in vivo*, but also described the function of the cytoplasmic *lincRNA-EPS* in modulating PKR-STAT1-dependent antiviral ISGs.

It is a good suggestion to investigate the effect of *lincRNA-EPS* on IFN-β production and IFNAR downstream signaling by using viral mimics instead of live virus. To eliminate the influence of inhibited virus replication by deficiency of *lincRNA-EPS* on the IFN-β production, we transfected the WT and *lincRNA-EPS*$^{-/-}$ iBMMs with polyI:C to trigger the IFN-β production and IFNAR downstream

signaling. As the results shown in the Fig EV5A and Fig 5I, polyI:C induced less IFN-β transcripts in the *lincRNA-EPS*$^{-/-}$ *iBMMs* (Fig EV5A), despite not as dramatic as that in VSV infection experiment (Fig 5A and B). The Western blot results shown in Fig 5I also supported the conclusion that knockout of *lincRNA-EPS* activate stronger IFNAR downstream signaling independent of IFN-β. Moreover, although we failed to get the detectable phosphorylation of IRF3 in the polydA:dT-transfected iBMMs, higher induction of pSTAT1, IFIT2, but less pTBK1 in the *lincRNA-EPS*$^{-/-}$ cells were also consistent with the conclusion obtained from the VSV-infected iBMMs (data were shown as below).

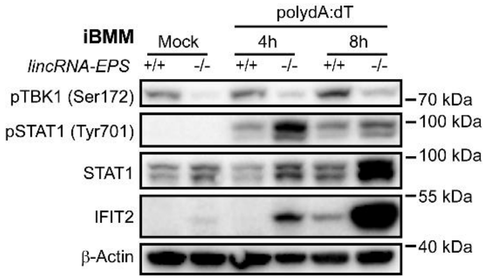


*2- The link with PKR is tenuous. This reviewer notes that there are an estimated 11 copies of EPS RNA per cell (based on the Cell paper cited above), and it is hard to imagine that these molecules would be naturally interacting with PKR when they are principally bound to nuclear chromatin (this needs to be discussed). Critically, most of the data linking PKR is based on RNAi with a single siRNA. Given the off-target effects of RNAi, it is essential to have at least two independent siRNAs targeted to the same target and showing the same phenotype to support any conclusions made. The theory of the authors does not explain how in the absence of infection, the ISG (STAT1 targets) they picked would be induced by the PKR-STAT1 axis (noting that STAT1-P is entirely absent in mock cells Fig 5H). Critically, if the PKR-STAT1 axis is really involved, then RNAi of STAT1 should also decrease the basal expression of selected ISGs basally engaged in EPS cells.*

**Response**: Thanks for your comments and insight questions.

1) According to the previous study (C. Liu, 2019 ***Cell***. PMID: 31031002), several copies of lncRNA are sufficient to suppress PKR activation. There are only 6 copies per cell of *circPOLR2A* in Hela cells. However, endogenous *circPOLR2A* suppresses PKR activation significantly (Figure 5 of the PMID: 31031002). Reduced *circPOLR2A* to ~1 copy per cell during polyI:C stimulation still inhibits the PKR activation (Figure 5 of the PMID: 31031002). 20-30% of *lincRNA-EPS* is located in

the cytoplasm (Fig 1J) and there are about 23 copies per cell of *lincRNA-EPS* in the iBMMs. Therefore, we think it is possible that cytoplasmic *lincRNA-EPS* is sufficient to interact with PKR, block viral RNA, and thus suppress host antiviral immunity.

2) To eliminate the potential off-target effect of RNAi, we designed two non-overlapping siRNA targeting PKR. Results from both siPKR consistently suggest that upregulation of antiviral ISGs in the *lincRNA-EPS*$^{-/-}$ iBMMs is required PKR (Figs 7C-H, EV5D-E).

3) Although pSTAT1 is undetectable in the mock WT and *lincRNA-EPS*$^{-/-}$ iBMMs, we still believe that the PKR-STAT1 signaling maintains basal level of ISG expression. Low level type I IFN and pSTAT1 maintains multiple basal ISG expression such as *Mx1* and *IFIT2* genes (PMID:30517866). We performed the experiment as you suggested, and found that knockdown of STAT1 also significantly decrease the basal expression of selected ISGs such as *Mx1* and *Oas2* in the *lincRNA-EPS*$^{-/-}$ iBMMs (data has been shown as below). It will be more convincing if the *lincRNA-EPS*$^{-/-}$*STAT1*$^{-/-}$ cells are available to verify these RNAi results in future.

*3-*

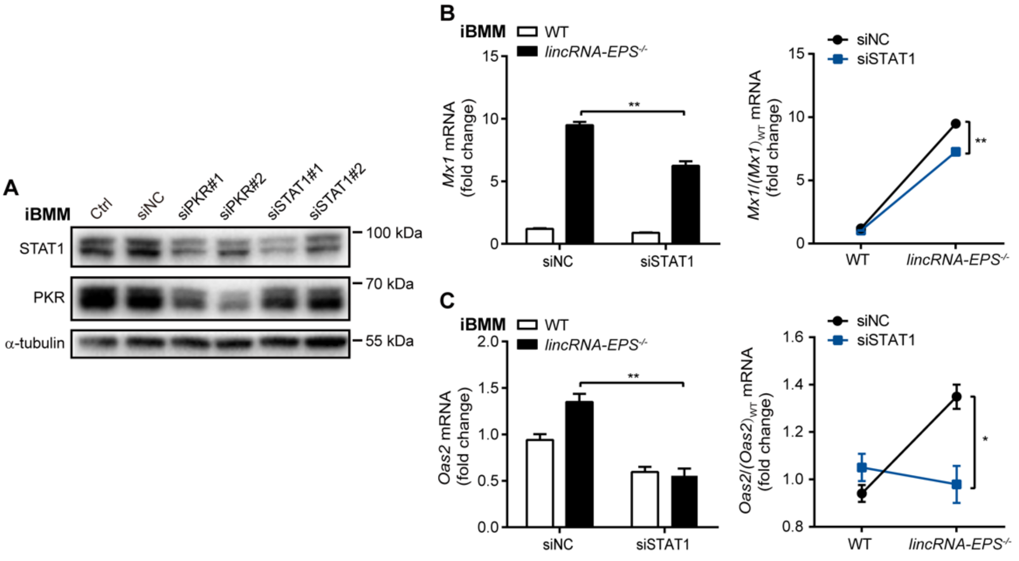


*The authors entirely omit the concept presented in the Atianand paper that EPS Maintains a Repressed Chromatin State at selected IRGs (including key antiviral genes) - which directly explains why some antiviral genes are basally up-regulated in EPS KO cells. Which genes would be PKR-STAT1 regulated and which genes would be hnRNPL-EPS controlled??? Since many antiviral genes such as RSAD2 and IFIT1 were found to be regulated by the hnRNPL-EPS axis it is hard to distinguish the antiviral effects of the proposed PKR-STAT1 axis (but this is an essential thing to perform experimentally to substantiate the overall point of the paper).*

**Response**: Thanks for your questions and suggestions. We agree that knockout of *lincRNA-EPS* changes the repressed chromatin state to a more accessible chromatin state at the promoters of selected IRGs including key antiviral genes. However, it is well accepted that accessibility of promoter DNA is not sufficient to drive gene transcription (PMID: 31511305 and 23595227). Our current study describes the cytoplasmic function of *lincRNA-EPS* in the regulation of antiviral immunity. The previous study by Atianand et al. (PMID: 27315481) demonstrates the nuclear function of *lincRNA-EPS* during inflammatory responses, which also shows that the basal expression of several antiviral ISGs including *Mx1* and *OAS2* are upregulated in the *lincRNA-EPS*$^{-/-}$ BMDMs (Fig 5F). Therefore, it is possible that cytoplasmic and nuclear *lincRNA-EPS* synergistically suppresses ISG expression at both resting and viral infected conditions. Nuclear *lincRNA-EPS*-hnRNPL complex determines chromatin accessibility of the ISG promoters, and the cytoplasmic *lincRNA-EPS*-PKR-STAT1 signaling axis controls ISG transcription. Although knockdown of PKR significantly downregulates STAT1 activation and ISGs induction. However, knockout of *lincRNA-EPS* makes it much easier to access the promoters of ISG by STAT1 and thus low-level activation of the PKR-STAT1 signaling is sufficient to induce ISG transcription in the *lincRNA-EPS*$^{-/-}$ iBMMs.

We performed an RNA-Seq and analyzed the transcriptome of the resting WT and *lincRNA-EPS*$^{-/-}$ iBMMs transfected with negative control and siPKR. Knockdown of PKR significantly downregulated numerous ISGs expression in both WT and *lincRNA-EPS*$^{-/-}$ iBMMs. However, the fold changes of ISG expression between siNC and siPKR groups are not significant (Appendix Figure S1 of the revised manuscript). Therefore, we think that hnRNPL-EPS axis and PKR-STAT1 axis synergistically control ISGs expression but not work separately.

Minor:

*1-Fig 2A/B... what is the biological relevance of a 12-fold increase of EPS RNA?*

**Response**: Thanks for your insight questions. Given that the fold change of *lincRNA-EPS* transcript during viral infection is between 2 to 20 folds (Fig 1A) and

the variation of *lincRNA-EPS* expression between macrophages is less than 20 folds (Fig EV1D), we chose moderate overexpression rather than the high overexpression to investigate the role of *lincRNA-EPS* during viral infection. Although we can artificially overexpress *lincRNA-EPS* over 100 folds, moderate upregulation is more reasonable to study the physiological and pathophysiological function of *lincRNA-EPS*.

*2-Fig 5F/G is missing color keys*

**Response**: Thanks for your reminding. We have added the color keys in Fig 5G of the revised manuscript.

*3-Why are you using immortalized BMDMs from EPS$^{-/-}$ mice (which end up being similar to your RAW cells) and not primary cells?*

**Response**: Thanks for your insight question. Actually, we have tested the function of *lincRNA-EPS* during viral infection by using several macrophages including two macrophage cell lines (RAW264.7 and iBMMs) and two primary macrophages (PM and BMDM). RAW264.7 and iBMMs always showed the robust and consistent phenotypes. Moreover, it is much easier for gene overexpression, knockdown, and viral infection in the iBMMs than the primary macrophages. Therefore, we used iBMMs more often than primary macrophages. However, we have confirmed several key experiments by using both PMs (Fig 3F and Fig 4D) and primary BMDMs (Fig EV4A) to make our study more physiological relevance. Based on our finding, it is likely that more copies of *lincRNA-EPS* in the iBMMs makes us easier to studies the function of *lincRNA-EPS* (Fig EV1D).

*4-Fig 6F should be relative to each siRNA (siPKR increased viral load in WT so cannot be 1).*

**Response**: Thanks for your suggestion. The increased viral load in the siPKR groups was shown in Fig 6E. However, in order to indicate the decrease rate of VSV titer in the siNC and siPKR groups, we used the parameter (VSV titer)/(VSV titer)$_{WT}$. (VSV titer)$_{WT}$/(VSV titer)$_{WT}$ should be 1 and (VSV titer)$_{ko}$/(VSV titer)$_{WT}$ should be less than

1. Results shown in Fig 6F could reflect the less decrease rate of viral load in the siPKR *lincRNA-EPS*$^{-/-}$ iBMMs than the WT cells.

=============================================================

Referee #2:

*lincRNA-EPS has been previously characterized as an immunoregulatory lncRNA that restrains inflammatory responses, although its role in antiviral immunity is unknown. Here they demonstrate that lincRNA-EPS as a negative regulator of host antiviral responses. They report that lincRNA-EPS is downregulated during RNA virus infection, to allow an adequate anti-viral response. The authors demonstrate that LincRNA-EPS binds PKR to block its interaction with viral RNA. Blockade of the PKR-viral RNA interaction impairs activation of STAT1 and ISG expression rendering mice more susceptible to RNA virus infection. The results are interesting and add to our knowledge of lncEPS and lncRNA dependent regulation of antiviral immunity more broadly.*

*There is an overreliance on immortalized cells and overexpression systems. The paper would be strengthened if some of the key observations could be validated in primary cells and an endogenous interaction of lincRNA-EPS and PKR examined.*

**Response**: We appreciate your comments and suggestions, which significantly improve the value of our study. As you suggested, we have performed the experiments by using both primary peritoneal macrophages and BMDMs. These results are shown in revised Fig 3F, 4D, and EV4A. The endogenous interaction of *lincRNA-EPS* and PKR were confirmed in RAW264.7 cells as shown in Fig 6C of the revised manuscript. Point-by-point response to your individual question has been provided as below.

Major comments:

*1. Figure 1J should include an analysis of absolute copy number of lincRNA-EPS. RNA FISH analysis could also be highly informative here.*

**Response**: Thanks for your suggestion. We have measured and calculated the absolute copy number of *lincRNA-EPS* transcript in the cell types that we used by RT-qPCR. As shown in Fig EV1D, RAW264.7 and iBMMs express about 63 and 23 copies *lincRNA-EPS* per cell, respectively, higher than BMDM (~8 copies/cell). Based on our finding, it is likely that more abundance of *lincRNA-EPS* in the iBMMs makes us easier to study the function of *lincRNA-EPS*.

*2. Throughout the figures are 1 experiment. Instead, they should include the mean of all independent experiments performed.*

**Response**: Thanks for reminding us to check the data analyzing and statistics. Actually, we performed most of experiments with biological triplicates, which should be considered as three independent experiments. In some cases, we used one representative results of three independent experiments. We have included the mean of all independent experiments performed and corrected the description in the figure legends of the revised manuscript.

*3. It's unclear why immortalized cells were used in Figure 3-5 rather than primary BMDM from lincRNA-EPS deficient mice and WT littermate controls. The main findings of these figures should be repeated using primary BMDMs.*

**Response**: Thanks for your question and valuable suggestion. We have repeated the VSV infection experiments in the primary PM and BMDM, and get the consistent phenotype as in the iBMMs and RAW264.7 cells (Figs 3F, 4D, and EV4A of the revised manuscript). Moreover, as the results shown in Fig 3I-J and Fig EV4B-C, we confirmed the conclusions from iBMMs and primary macrophages experiments by checking the viral load, IFN-β, and ISGs expression in livers and lungs from VSV-infected mice.

*4. The decrease in viral titer in the serum of lincRNA-EPS mice (Figure 3J) is very modest when compared to WT and is not consistent with the robust phenotype observed in Figure 3G. It would be beneficial to assess the viral titer in the liver and lungs after infection.*

**Response**: Thanks for your comments which will greatly improve the significance of our study. In the survival rate experiment, we injected lethal dose of VSV intravenously ($1\times10^8$ PFU/g) to cause most of WT mice dead at 24 h post infection. However, in order to get enough samples, especially the serum sample in the VSV-infected WT mice, we decrease the infection viral titer to sub-lethal dose by intraperitoneally injection, which may partially explain the modest phenotype in the serum viral load when comparing to the survival results. In the revised manuscript, we

repeated this key experiment by injecting VSV with titer of $6\times10^{7}$ PFU/g intravenously and get more robust phenotype on viral load (left panel of Fig 3I). In addition, the viral titer in the livers and lungs are checked and the results are shown in revised Fig 3I (middle and right panels).

*5. Figure 4 should include protein levels of at least some representative ISGs.*

**Response**: Thanks for your suggestions. As shown in the Fig 5H and I, we added the protein bands of antiviral ISG IFIT2 during iBMMs infected with VSV or transfected with polyI:C. Both IFIT2 transcripts and proteins levels are higher induced in *lincRNA-EPS*$^{-/-}$ iBMMs than WT cells during antiviral immunity.

*6. The authors should include a full list of lincRNA-EPS interacting proteins identified by mass spectrometry. Additional controls such as the use of biotinylated lincRNA-EPS probes in lincRNA-EPS deficient BMDMs would further strengthen this part of the study.*

**Response**: Thanks for your suggestions. We have provided Table EV1 which listed *lincRNA-EPS* interacting proteins from the marked protein band as indicated in Fig 6A. The positive control gene hnRNPL and target gene PKR were yellow highlighted in Table EV1. We also pulled down the target protein PKR from both WT and *lincRNA-EPS*$^{-/-}$ iBMMs by using biotinylated *lincRNA-EPS* (revised Fig 6B). Because of the slightly elevated PKR and more dissociated PKR from PKR-*lincRNA-EPS* complex, much more PKR was pulled down by the biotinylated *lincRNA-EPS* in the *lincRNA-EPS*$^{-/-}$ iBMMs than in the WT cells. In addition, biotinylated polyI:C, another potent ligand of PKR, also pulled down more PKR in the *lincRNA-EPS*$^{-/-}$ iBMMs than in the WT cells.

*7. Figure 7C-G demonstrate upregulated expression of ISGs in mock cells deficient in lincRNA-EPS when compared to WT cells. However, no phospo-STAT1 was detected in lincRNA-EPS KI cells (Figure 5H). The authors should explain this discrepancy.*

**Response**: Thanks for your comments and suggestion. We think that the basal level of phosphorylated STAT1 is difficult to be detected because of the limited sensitivity of pSTAT1 antibody. However, basal pSTAT1 exists since the constitutive expression of

low-level type I IFNs. Actually, basal type I IFN or pSTAT1 maintains multiple ISG expression such as *Mx1* and *IFIT2* genes (PMID:30517866).

*8. Figure 7I: a better explanation of the proposed mechanism including subcellular localization should be provided.*

**Response**: Thanks for your suggestion. We have added more explanation including subcellular locations in the last paragraph of Results (line 302-305) and Discussion section (line 322-334). We also clearly labeled the cytoplasm and nucleus in the revised Fig7I. In this study, we mainly describe the cytoplasmic function of *lincRNA-EPS*, which is different from the nuclear function of *lincRNA-EPS* during inflammatory responses demonstrated by Atianand *et al.* (PMID: 27315481).

Minor comments:

*1. Figure 4A: The authors should provide a heatmap demonstrating a full list of genes and pathways detected by the assay.*

**Response**: Thanks for your suggestion. We have provided the heatmaps for ISGs (Fig 4B of the revised manuscript) and the DEGs belong to the top three pathways of GSEA (Fig 4A and Fig EV3A of the revised manuscript).

*2. Figure 5F is missing a legend.*

**Response**: Thanks for your reminding. We have added the legend in the revised Fig 5G.

*3. Figure 6D should include detection of PKR in naïve cells as an additional negative control.*

**Response**: Thanks for your suggestion. We have included an additional control (siRNA without transfection reagent, labeled as "Ctrl") in the Fig EV5B of the revised manuscript. In addition, the knockdown efficiency of two siRNAs targeting PKR is clearly shown in the revised Fig EV5B.

*4. Figure 7A: Can the author explain why total STAT1 is downregulated in PKR deficient cells?*

**Response**: Thanks for your question. We used two non-overlapping siRNA targeting PKR to eliminate the off-target effect of RNAi, and observed the downregulation of

STAT1 in both knockdown cells (Fig EV5B). Overexpression of PKR in MEF cells also upregulated STAT1 expression and STAT1 phosphorylation (Fig EV5C). Given that STAT1 is also an ISG, it is possible that PKR-STAT1 signaling proposed in Fig 7I (left panel) also controls STAT1 itself expression. However, the detail mechanism that explain how PKR regulates STAT1 activation needs to be further studied. According to our published and unpublished studies, we think that multiple kinases work similar as Jak1 which directly activates STAT1.

==========================================================

Referee #3:

*In this study, Zhu et al. describe a role of LincRNA-EPS in the negative regulation of STAT1-triggered type I IFN responses. PKR is well-known as a nucleic acid receptor of viral dsRNA. PKR activity needs to be restricted in the resting state to prevent autoimmune activation. The authors claim that lincRNA-EPS binds to PKR and antagonizes the viral RNA-PKR interaction and then inhibits PKR-induced STAT1 activation, resulted in the suppression of downstream ISGs production. Virus infection significantly decreases LincRNA-EPS expression and subsequently removes the restrictions of PKR activation. Therefore, this study helps us to understand the self-limiting of the innate immune response. Overall, the findings are novel and interesting. The experiments are well performed, and the manuscript is fluent and generally well organized. However, some concerns mentioned below must be addressed before the manuscript can be considered for publication.*

**Response**: We appreciate your positive comments and valuable suggestions. Point-by-point response to your individual question has been provided as below.

Major comments:

*1.As the author describes that PKR is required for phosphorylation and activation of STAT1 under the response to IFN-γ and LPS, but PKR or LincRNA-EPS inhibition greatly changed the total protein level of STAT1(Fig5H, Fig7A and 7B). Activation and phosphorylation of STAT1 should not affect its total protein level, is there any other mechanism for regulation of STAT1?*

**Response**: Thanks for your question. According to our conclusions, knockout of *lincRNA-EPS* removes the restrictions of PKR activation, which may maintain basal STAT1 activation and ISG induction via the PKR-STAT1 signaling. Given that STAT1 itself is an ISG (PMID: 19478064), it is possible that PKR-STAT1 signaling proposed in Fig 7I (left panel) also controls STAT1 itself expression. Therefore, it is reasonable to observe elevated and downregulated STAT1 expression in the *lincRNA-EPS*$^{-/-}$ and PKR knockdown iBMMs, respectively. In addition, we have confirmed that overexpression of PKR in MEF cells also upregulates STAT1 expression and this induction is suppressed by *lincRNA-EPS* (Fig EV5C), which is consistent with the results observed the in the *lincRNA-EPS*$^{-/-}$ and PKR knockdown cells.

*2.In Fig 7C, 7E and 7G, WT-siPKR almost blocked VSV infection-induced ISGs expression, but LincRNA-EPS-/--siPKR greatly enhanced ISGs expression compared with WT-siPKR. Therefore, PKR knockdown could not change the positive effect of lincRNA-EPS knockout on ISG formation, these results strongly support the existence of other mechanisms that regulate JAK-STAT signaling by LincRNA-EPS. The authors should use IFN-β stimulation instead of VSV infection to investigate whether this phenomenon is caused by viral infection specificity.*

**Response**: Thanks for your comments and suggestions. Our current study describes the cytoplasmic function of *lincRNA-EPS* in the regulation of antiviral immunity. A previous study by Atianand et al. (PMID: 27315481) has demonstrated the nuclear function of *lincRNA-EPS* during inflammatory responses, which also shows that the basal expression of several antiviral ISGs including Mx1 and OAS2 are upregulated in the *lincRNA-EPS*$^{-/-}$ BMMs (Fig 5F). Therefore, it is possible that cytoplasmic and nuclear lincRNA-EPS synergistically suppresses ISG expression. Nuclear *lincRNA-EPS*-hnRNPL complex determines chromatin accessibility of the ISG promoters, and the cytoplasmic *lincRNA-EPS*-PKR-STAT1 signaling axis controls ISG transcription. Knockdown of PKR significantly downregulates STAT1 activation and ISGs induction. However, knockout of *lincRNA-EPS* makes it much easier to access the promoters of ISG by STAT1 and thus low-level activation of the

PKR-STAT1 signaling is sufficient to induce ISG transcription in the *lincRNA-EPS*$^{-/-}$ iBMMs.

We don't think the inhibitory function *lincRNA-EPS* is viral infection specific, since the results based on viral mimics polyI:C shows viral mimics also compete with *lincRNA-EPS* to interacts with PKR (revised Fig 6B and I). Similar as VSV infection, polyI:C also induced more pSTAT1 and ISG in the *lincRNA-EPS*$^{-/-}$ iBMMs than in the WT cells (revised Fig 5I).

*3.The H3K4me3 is associated with high transcription activity. However, LincRNA-EPS knockout does not affect the IFN-β induced H3K4me3 of ISGs in the activation state but not the rest state(Fig 5G and 5H). What are the reasons for the different effect？*

**Response**: Thanks for your question and pointing out these unconvincing results. It is possible that too high concentration of IFN-β treatment (500 U/mL) lead to the sutured induction of H3K4me3 interacting with ISG promoters, which caused almost no difference between *lincRNA-EPS*$^{-/-}$ and WT groups in the activation state. We repeated this experiment by using optimized IFN-β concentration or viral RNA mimics polyI:C. As shown in Fig 5G of the revised manuscript, the transcription activities of ISG were elevated in the *lincRNA-EPS*$^{-/-}$ iBMMs than the WT group, both in the resting and polyI:C-triggered activation states.

*4.The authors claim that lincRNA-EPS regulates STAT1 activation and subsequently controls ISGs expression. However, the study only examined the effects of lincRNA-EPS on virus replication and tissue damage in vivo. Whether lincRNA-EPS could control ISGs expression in tissues (such as lung, spleen or liver) or serum?*

**Response**: Thanks for your comments and suggestions. As suggested, we have repeated the VSV infection experiment *in vivo,* and measured the ISGs level as well as the viral load in tissues of liver and lung. Less viral load, lower IFN-β, but higher ISG expression were observed in livers and lungs from the *lincRNA-EPS*$^{-/-}$ mice than the WT mice (Figs 3I, J, EV4B and C of the revised manuscript), which indicate that *lincRNA-EPS* controls host antiviral immunity against VSV by regulating ISG expression in tissues.

*5.In Fig 6H and 6I, lincRNA-EPS overexpression competitively binds virus RNA, which in turn inhibits PKR activation, but more evidence is needed to support this conclusion. Whether lincRNA-EPS knockout or knockdown could promote PKR binds RNA? Whether other dsRNA (such as poly(I: C) have similar phenomena to the VSV-RNA?*

**Response**: Thanks for your comments and suggestions. To investigate the effect of *lincRNA-EPS* deficiency on the interaction between PKR and viral RNA, we used biotinylated *lincRNA-EPS* and polyI:C to pull down the endogenous PKR protein. As shown in Fig 6B of the revised manuscript, we observed more PKR was pulled down by both biotinylated *lincRNA-EPS* and polyI:C from *lincRNA-EPS*$^{-/-}$ iBMMs cell lysates than the WT cell lysates. To further study whether lincRNA-EPS and polyI:C competitively binds with PKR, we transfected different doses of polyI:C into iBMM. *LincRNA-EPS*-interacted PKR protein was decreased in a dose-dependent manner after the cells transfected with polyI:C (Fig 6I of the revised manuscript), which suggest that *lincRNA-EPS* and other dsRNA such as polyI:C competitively binds with PKR.

Minor comments:

*1.The graphic symbols are missing in Fig5F and 5G.*

**Response**: Thanks for your reminding. We have added the graphic symbols in the revised Fig 5G.

*2.Some description of the background of VSV-N and G is required.*

**Response**: Thanks for your suggestion. We have added the background of VSV-N and G in the Results section (line 271-274).

*3.In Fig 3G, the survival percentage of the lincRNA-EPS knockout group did not appear to be calculated from 100%.*

**Response**: Thanks for your reminding. We have changed the line color and clearly shown that the survival percentage of *lincRNA-EPS*$^{-/-}$ group was calculated from 100% in the revised Fig 3G.

==========================================================

*Further comments from referee 1:*

*If the authors conducted an RNAseq experiment of EPS-WT and EPS-KO after PKR down-regulation, it may help provide insights into the claimed PKR/EPS-dependent ISG signature - which should only be a portion of the ISGs see in EPS-WT. KO or down-regulation of such PKR-dependent ISGs in the context of EPS-KO cells with infection would be the only direct way to link EPS-PKR targets and antiviral effects.*

**Response**: Thanks for your suggestion. We have conducted an RNA-seq experiment of resting WT and *lincRNA-EPS*$^{-/-}$ iBMMs after PKR down-regulation. However, we did not find specific genes regulated by hnRNPL-EPS axis or PKR-STAT1 axis. Knockdown of PKR significantly downregulated numerous ISGs expression in both WT and *lincRNA-EPS*$^{-/-}$ iBMMs. However, the fold changes of ISG expression between siNC and siPKR groups are not significant (Appendix Figure S1 of the revised manuscript). Therefore, we think that hnRNPL-EPS axis and PKR-STAT1 axis synergistically control ISGs expression but not work separately. More detail answer is in the 3rd questions of Reviewer#1.

Dear Prof. Ma,

Thank you for the submission of your revised manuscript. We have now received the enclosed reports from the referees that were asked to assess it. Referee 1 still has some minor suggestions that I would like you to incorporate before we can proceed with the official acceptance of your manuscript.

Referee 1 also assessed your reply to referee 2's concerns and noted that it is unclear whether technical or biological repeats were performed throughout the manuscript. This is a very important point. Technical repeats are performed in parallel in the context of one experiment, and biological repeats are independently performed experiments, on different days and ideally with different animals/cells/solutions/materials. Please make sure that all figure legends correctly state whether technical or biological repeats were performed. It is absolutely essential that the most important experiments in your study are performed at least 3 times independently, and the mean data of these independent experiments (plus statistical analyses) should be shown in the figures.

A few other editorial requests also need to be addressed:

- Please correct the subheading to "Disclosure and Competing Interests Statement" and confirm that your statement is in line with our journal policy: https://www.embopress.org/competing-interests

- All funding information also needs to be listed in our online manuscript system when you upload the final version of your manuscript. The online version is currently incomplete.

- If you like, you can change the APPENDIX figure into Figure EV6, as we do allow exceptionally 6 EV figures. Alternatively, you could also combine 2 EV figures to keep the maximum number of 5 EV figures.

- The EV figure legends need to be pasted to after the main figure legends in the main manuscript file.

I attach to this email a related manuscript file with comments by our data editors. Please address all comments in the final manuscript.

I would like to suggest a few changes to the title and abstract. Do you agree with the following:

LincRNA-EPS impairs host antiviral immunity by antagonizing viral RNA-PKR interaction

LincRNA-EPS is an important regulator in inflammation. However, the role of lincRNA-EPS in the host response against viral infection is unexplored. Here, we show that lincRNA-EPS is downregulated in macrophages infected with different viruses including VSV, SeV, and HSV-1. Overexpression of lincRNA-EPS facilitates viral infection, while downregulation of lincRNA-EPS protects the host against viral infection in vitro and in vivo. lincRNA-EPS-/- macrophages show elevated expression of antiviral interferon-stimulated genes (ISGs), such as Mx1, Oas2, and Ifit2 at both basal and inducible levels. However, IFN-b, the key upstream inducer of these ISGs, is downregulated in lincRNA-EPS-/- macrophages compared with control cells. RNA pulldown and mass spectrometry results indicate that lincRNA-EPS binds to PKR and antagonizes the viral RNA-PKR interaction. PKR activates STAT1 and induces antiviral ISGs independent of IFN-I induction. LincRNA-EPS inhibits PKR-STAT1-ISGs signaling and thus facilitates viral infection. Our study outlines an alternative antiviral pathway, with downregulation of lincRNA-EPS promoting the induction of PKR-STAT1-dependent ISGs, and reveals a potential therapeutic target for viral infectious diseases.

EMBO press papers are accompanied online by A) a short (1-2 sentences) summary of the findings and their significance, B) 2-3 bullet points highlighting key results and C) a synopsis image that is exactly 550 pixels wide and 200-600 pixels high (the height is variable). You can either show a model or key data in the synopsis image. Please note that text needs to be readable at the final size. Please send us this information along with the revised manuscript.

I look forward to seeing a new revised version of your manuscript as soon as possible. Please use this link to submit your revision: https://embor.msubmit.net/cgi-bin/main.plex

Best regards,
Esther

Esther Schnapp, PhD
Senior Editor
EMBO reports

Referee #1:

The authors have carefully addressed my comments in their rebuttal.
However, the following points should be added to the manuscript itself.
1) The response to point 2 regarding the plausibility of EPS to bind to PKR given its low expression (about the comparison with CircPOLR2A) should be mentioned in the discussion.
2) Figure with siSTAT1 and its impact on MX1 and Oas2 should be added to the manuscript and discussed - as it directly links STAT1 and the observations.

Referee #3:

The authors have addressed all my concerns. I have no further comments.

Dear Dr. Esther Schnapp,

We are submitting our revised manuscript entitled "LincRNA-EPS impairs host antiviral immunity by antagonizing viral RNA interacting with PKR" (EMBOR-2021-53937V3) to ***EMBO Reports*** for consideration of publication.

We appreciate all the suggestions from you and referee 1. We have corrected the subheading to "Disclosure and Competing Interests Statement", listed all funding information in the online manuscript system, changed the APPENDIX figure into Figure EV6, pasted all the EV figure legends after the main figure legends in the main manuscript file. We have also revised the title and abstract as your great suggestions. In addition, the experimental repeats have been checked and described in the figure legends.

**The short summary of the findings and their significance:**

The long non-coding RNA lincRNA-EPS binds to PKR in cytoplasm of macrophages, antagonizes viral RNA-PKR interaction, and thus impairs PKR-STAT1-dependent host antiviral immunity.

**Bullet points:**

- LincRNA-EPS is downregulated by host antiviral immunity
- Knockout of lincRNA-EPS protects host against viral infection
- LincRNA-EPS antagonizes viral RNA-PKR interaction
- LincRNA-EPS impairs PKR-STAT1-dependent induction of antiviral ISGs

A synopsis image with proper size and resolution has been submitted online.

**Responses to the suggestions from Referee #1:**

1) *The response to point 2 regarding the plausibility of EPS to bind to PKR given its low expression (about the comparison with CircPOLR2A) should be mentioned in the discussion.*

**Response:** We have discussed the plausibility of cytoplasmic lincRNA-EPS binding to PKR by comparing the RNA copies per cell between lincRNA-EPS and CircPOLR2A (line 359-364 of the revised manuscript, yellow highlighted).

2) *Figure with siSTAT1 and its impact on MX1 and Oas2 should be added to the manuscript and discussed - as it directly links STAT1 and the observations.*

**Response:** The results about siSTAT1 and its impact on MX1 and Oas2 have been shown in the Figure EV5G. We have also described and discussed these data (line 298-301 of the revised manuscript, yellow highlighted).

We think that we have addressed all the concerns raised by you and Referee 1. Thank you very much for your consideration. We are looking forward to hearing from you.

With my best regards

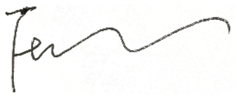

Feng Ma, M.D.

Institute of Systems Medicine, Chinese Academy of Medical Sciences

100 Chongwen Road, Suzhou Industry Park

Suzhou215123, Jiangsu, China

Prof. Feng Ma
Chinese Academy of Medical Sciences
800 Chongwen Road
Suzhou, Jiangsu 215123
China

Dear Prof. Ma,

I am very pleased to accept your manuscript for publication in the next available issue of EMBO reports. Thank you for your contribution to our journal.

At the end of this email I include important information about how to proceed. Please ensure that you take the time to read the information and complete and return the necessary forms to allow us to publish your manuscript as quickly as possible.

As part of the EMBO publication's Transparent Editorial Process, EMBO reports publishes online a Review Process File to accompany accepted manuscripts. As you are aware, this File will be published in conjunction with your paper and will include the referee reports, your point-by-point response and all pertinent correspondence relating to the manuscript.

If you do NOT want this File to be published, please inform the editorial office within 2 days, if you have not done so already, otherwise the File will be published by default [contact: emboreports@embo.org]. If you do opt out, the Review Process File link will point to the following statement: "No Review Process File is available with this article, as the authors have chosen not to make the review process public in this case."

Should you be planning a Press Release on your article, please get in contact with emboreports@wiley.com as early as possible, in order to coordinate publication and release dates.

Thank you again for your contribution to EMBO reports and congratulations on a successful publication. Please consider us again in the future for your most exciting work.

Yours sincerely,

Esther Schnapp, PhD
Senior Editor
EMBO reports

**************************************************************************************

THINGS TO DO NOW:

Please note that you will be contacted by Wiley Author Services to complete licensing and payment information. The required 'Page Charges Authorization Form' is available here: https://www.embopress.org/pb-assets/embo-site/er_apc.pdf

You will receive proofs by e-mail approximately 2-3 weeks after all relevant files have been sent to our Production Office; you should return your corrections within 2 days of receiving the proofs.

Please inform us if there is likely to be any difficulty in reaching you at the above address at that time. Failure to meet our deadlines may result in a delay of publication, or publication without your corrections.

All further communications concerning your paper should quote reference number EMBOR-2021-53937V3 and be addressed to emboreports@wiley.com.

Should you be planning a Press Release on your article, please get in contact with emboreports@wiley.com as early as possible, in order to coordinate publication and release dates.

## EMBO Press Author Checklist


| | |
|---|---|
| Corresponding Author Name: Feng Ma | |
| Journal Submitted to: EMBO Reports | |
| Manuscript Number: EMBOR-2021-53937V2 | |


**USEFUL LINKS FOR COMPLETING THIS FORM**
The EMBO Journal - Author Guidelines
EMBO Reports - Author Guidelines
Molecular Systems Biology - Author Guidelines
EMBO Molecular Medicine - Author Guidelines

**Reporting Checklist for Life Science Articles (updated January 2022)**
This checklist is adapted from Materials Design Analysis Reporting (MDAR) Checklist for Authors. MDAR establishes a minimum set of requirements in transparent reporting in the life sciences (see Statement of Task: 10.31222/osf.io/9sm4x). Please follow the journal's guidelines in preparing your manuscript.
**Please note that a copy of this checklist will be published alongside your article.**

**Abridged guidelines for figures**
**1. Data**
The data shown in figures should satisfy the following conditions:
- the data were obtained and processed according to the field's best practice and are presented to reflect the results of the experiments in an accurate and unbiased manner.
- ideally, figure panels should include only measurements that are directly comparable to each other and obtained with the same assay.
- plots include clearly labeled error bars for independent experiments and sample sizes. Unless justified, error bars should not be shown for technical replicates.
- if n<5, the individual data points from each experiment should be plotted. Any statistical test employed should be justified.
- Source Data should be included to report the data underlying figures according to the guidelines set out in the authorship guidelines on Data Presentation.

**2. Captions**
Each figure caption should contain the following information, for each panel where they are relevant:
- a specification of the experimental system investigated (eg cell line, species name).
- the assay(s) and method(s) used to carry out the reported observations and measurements.
- an explicit mention of the biological and chemical entity(ies) that are being measured.
- an explicit mention of the biological and chemical entity(ies) that are altered/varied/perturbed in a controlled manner.
- the exact sample size (n) for each experimental group/condition, given as a number, not a range;
- a description of the sample collection allowing the reader to understand whether the samples represent technical or biological replicates (including how many animals, litters, cultures, etc.).
- a statement of how many times the experiment shown was independently replicated in the laboratory.
- definitions of statistical methods and measures:
  - common tests, such as t-test (please specify whether paired vs. unpaired), simple χ2 tests, Wilcoxon and Mann-Whitney tests, can be unambiguously identified by name only, but more complex techniques should be described in the methods section;
  - are tests one-sided or two-sided?
  - are there adjustments for multiple comparisons?
  - exact statistical test results, e.g., P values = x but not P values < x;
  - definition of 'center values' as median or average;
  - definition of error bars as s.d. or s.e.m.

**Please complete ALL of the questions below.**
**Select "Not Applicable" only when the requested information is not relevant for your study.**

### Materials

| Newly Created Materials | Information included in the manuscript? | In which section is the information available? (Reagents and Tools Table, Materials and Methods, Figures, Data Availability Section) |
|---|---|---|
| New materials and reagents need to be available; do any restrictions apply? | Yes | Materials and Methods |

| Antibodies | Information included in the manuscript? | In which section is the information available? (Reagents and Tools Table, Materials and Methods, Figures, Data Availability Section) |
|---|---|---|
| For **antibodies** provide the following information:<br>- Commercial antibodies: RRID (if possible) or supplier name, catalogue number and or/clone number<br>- Non-commercial: RRID or citation | Yes | Materials and Methods |

| DNA and RNA sequences | Information included in the manuscript? | In which section is the information available? (Reagents and Tools Table, Materials and Methods, Figures, Data Availability Section) |
|---|---|---|
| **Short novel DNA or RNA including primers, probes**: provide the sequences. | Yes | Materials and Methods |

| Cell materials | Information included in the manuscript? | In which section is the information available? (Reagents and Tools Table, Materials and Methods, Figures, Data Availability Section) |
|---|---|---|
| **Cell lines:** Provide species information, strain. Provide accession number in repository **OR** supplier name, catalog number, clone number, and/**OR** RRID. | Yes | Materials and Methods |
| **Primary cultures:** Provide species, strain, sex of origin, genetic modification status. | Not Applicable | |
| Report if the cell lines were recently **authenticated** (e.g., by STR profiling) and tested for mycoplasma contamination. | Not Applicable | |

| Experimental animals | Information included in the manuscript? | In which section is the information available? (Reagents and Tools Table, Materials and Methods, Figures, Data Availability Section) |
|---|---|---|
| **Laboratory animals or Model organisms:** Provide species, strain, sex, age, genetic modification status. Provide accession number in repository **OR** supplier name, catalog number, clone number, **OR** RRID. | Yes | Materials and Methods |
| **Animal observed in or captured from the field:** Provide species, sex, and age where possible. | Yes | Materials and Methods |
| Please detail **housing and husbandry conditions**. | Yes | Materials and Methods |

| Plants and microbes | Information included in the manuscript? | In which section is the information available? (Reagents and Tools Table, Materials and Methods, Figures, Data Availability Section) |
|---|---|---|
| **Plants:** provide species and strain, ecotype and cultivar where relevant, unique accession number if available, and source (including location for collected wild specimens). | Not Applicable | |
| **Microbes:** provide species and strain, unique accession number if available, and source. | Yes | Materials and Methods |

| Human research participants | Information included in the manuscript? | In which section is the information available? (Reagents and Tools Table, Materials and Methods, Figures, Data Availability Section) |
|---|---|---|
| If collected and within the bounds of privacy constraints report on age, sex and gender or ethnicity for all study participants. | Not Applicable | |

| Core facilities | Information included in the manuscript? | In which section is the information available? (Reagents and Tools Table, Materials and Methods, Figures, Data Availability Section) |
|---|---|---|
| If your work benefited from core facilities, was their service mentioned in the acknowledgments section? | Not Applicable | |

### Design

| Study protocol | Information included in the manuscript? | In which section is the information available? (Reagents and Tools Table, Materials and Methods, Figures, Data Availability Section) |
|---|---|---|
| If study protocol has been **pre-registered, provide DOI in the manuscript**. For clinical trials, provide the trial registration number **OR** cite DOI. | Not Applicable | |
| Report the **clinical trial registration number** (at ClinicalTrials.gov or equivalent), where applicable. | Not Applicable | |

| Laboratory protocol | Information included in the manuscript? | In which section is the information available? (Reagents and Tools Table, Materials and Methods, Figures, Data Availability Section) |
|---|---|---|
| Provide DOI OR other citation details if **external detailed step-by-step protocols** are available. | Not Applicable | |

| Experimental study design and statistics | Information included in the manuscript? | In which section is the information available? (Reagents and Tools Table, Materials and Methods, Figures, Data Availability Section) |
|---|---|---|
| Include a statement about **sample size** estimate even if no statistical methods were used. | Not Applicable | |
| Were any steps taken to minimize the effects of subjective bias when allocating animals/samples to treatment (e.g. **randomization procedure**)? If yes, have they been described? | Not Applicable | |
| Include a statement about **blinding** even if no blinding was done. | Not Applicable | |
| Describe **inclusion/exclusion criteria** if samples or animals were excluded from the analysis. Were the criteria pre-established?<br><br>If sample or data points were omitted from analysis, report if this was due to attrition or intentional exclusion and provide justification. | Not Applicable | |
| For every figure, are **statistical tests** justified as appropriate? Do the data meet the assumptions of the tests (e.g., normal distribution)? Describe any methods used to assess it. Is there an estimate of variation within each group of data? Is the variance similar between the groups that are being statistically compared? | Yes | Materials and Methods, Figures |

| Sample definition and in-laboratory replication | Information included in the manuscript? | In which section is the information available? (Reagents and Tools Table, Materials and Methods, Figures, Data Availability Section) |
|---|---|---|
| In the figure legends: state number of times the experiment was **replicated** in laboratory. | Yes | Figures |
| In the figure legends: define whether data describe **technical or biological replicates**. | Yes | Figures |

## Ethics

| Ethics | Information included in the manuscript? | In which section is the information available? (Reagents and Tools Table, Materials and Methods, Figures, Data Availability Section) |
|---|---|---|
| Studies involving **human participants**: State details of **authority granting ethics approval** (IRB or equivalent committee(s), provide reference number for approval. | Not Applicable | |
| Studies involving **human participants**: Include a statement confirming that **informed consent** was obtained from all subjects and that the experiments conformed to the principles set out in the WMA Declaration of Helsinki and the Department of Health and Human Services Belmont Report. | Not Applicable | |
| Studies involving **human participants:** For publication of **patient photos**, include a statement confirming that consent to publish was obtained. | Not Applicable | |
| Studies involving experimental **animals**: State details of **authority granting ethics approval** (IRB or equivalent committee(s), provide reference number for approval. Include a statement of compliance with ethical regulations. | Yes | Materials and Methods |
| Studies involving **specimen and field samples:** State if relevant **permits** obtained, provide details of authority approving study; if none were required, explain why. | Not Applicable | |

| Dual Use Research of Concern (DURC) | Information included in the manuscript? | In which section is the information available? (Reagents and Tools Table, Materials and Methods, Figures, Data Availability Section) |
|---|---|---|
| Could your study fall under dual use research restrictions? Please check biosecurity documents and list of **select agents and toxins** (CDC): https://www.selectagents.gov/sat/list.htm | Not Applicable | |
| If you used a select agent, is the security level of the lab appropriate and reported in the manuscript? | Not Applicable | |
| If a study is subject to dual use research of concern regulations, is the name of the **authority granting approval and reference number** for the regulatory approval provided in the manuscript? | Not Applicable | |

## Reporting

The MDAR framework recommends adoption of discipline-specific guidelines, established and endorsed through community initiatives. Journals have their own policy about requiring specific guidelines and recommendations to complement MDAR.

| Adherence to community standards | Information included in the manuscript? | In which section is the information available? (Reagents and Tools Table, Materials and Methods, Figures, Data Availability Section) |
|---|---|---|
| State if relevant guidelines or checklists (e.g., **ICMJE, MIBBI, ARRIVE, PRISMA**) have been followed or provided. | Not Applicable | |
| For **tumor marker prognostic studies,** we recommend that you follow the **REMARK** reporting guidelines (see link list at top right). See author guidelines, under 'Reporting Guidelines'. Please confirm you have followed these guidelines. | Not Applicable | |
| For **phase II and III randomized controlled trials**, please refer to the **CONSORT** flow diagram (see link list at top right) and submit the CONSORT checklist (see link list at top right) with your submission. See author guidelines, under 'Reporting Guidelines'. Please confirm you have submitted this list. | Not Applicable | |

## Data Availability

| Data availability | Information included in the manuscript? | In which section is the information available? (Reagents and Tools Table, Materials and Methods, Figures, Data Availability Section) |
|---|---|---|
| Have **primary datasets** been deposited according to the journal's guidelines (see 'Data Deposition' section) and the respective accession numbers provided in the Data Availability Section? | Yes | Materials and Methods, Data Availability Section |
| Were **human clinical and genomic datasets** deposited in a public access-controlled repository in accordance to ethical obligations to the patients and to the applicable consent agreement? | Not Applicable | |
| Are **computational models** that are central and integral to a study available without restrictions in a machine-readable form? Were the relevant accession numbers or links provided? | Not Applicable | |
| If publicly available data were reused, provide the respective **data citations in the reference list**. | Yes | |
